# Population Dynamics and Potential Distribution of the Four Endangered Mangrove Species in Leizhou Peninsula China

**DOI:** 10.3390/plants14213381

**Published:** 2025-11-05

**Authors:** Jianjian Huang, Bing Yang, Jie Chen, Suqing Liu, Xueying Wen, Yingchun Zhu, Kangyi Deng, Hui Zhu, Yuzhong Zheng, Qinghan Wu, Yongqin Zheng, Jean Wan Hong Yong, Fengnian Wu, Xiaolong Lan

**Affiliations:** 1 School of Life Sciences and Food Engineering, Hanshan Normal University, Chaozhou 521041, China; 2 East Guangdong Mangrove & Wetland Protection Centre, Hanshan Normal University, Chaozhou 521041, China; 3 College of Coastal Agricultural Sciences, Guangdong Ocean University, Zhanjiang 524088, China; 4 Department of Biosystems and Technology, Swedish University of Agricultural Sciences, 23456 Alnarp, Sweden; 5 Brightlands Future of Farming Institute, Faculty of Science and Engineering, Maastricht University, 5928 SX Venlo, The Netherlands

**Keywords:** mangrove, population dynamics, survival curve, environmental factors, conservation strategies

## Abstract

**Background**: Mangrove plants are a core component of coastal ecosystems, directly influencing biodiversity and shoreline stability. However, in recent years, due to the combined pressures of human activities and climate change, nearly half of the mangrove species in China are endangered and require urgent conservation measures. This study analyzed the population dynamics and stress factors affecting four rare and endangered mangrove species—*Lumnitzera racemosa*, *Ceriops tagal*, *Barringtonia racemosa*, and *Heritiera littoralis*—on the Leizhou Peninsula, providing scientific evidence for their conservation. **Methods**: Field surveys and plot investigations were conducted, with population dynamics and structure quantified using static life tables, survival rates, mortality rates, and disappearance curves. Additionally, the MaxEnt species distribution model and GIS technology were applied to predict the potentially suitable distribution areas. **Results**: The findings revealed that the population of *L. racemosa* exhibits an atypical pyramid structure, with few seedlings and constraining population growth potential. The *C. tagal* population follows an irregular pyramid structure, with abundant seedlings but fewer mature individuals, suggesting a rapid decline followed by stability. The *B. racemosa* population also follows an irregular pyramid structure, with many seedlings and a greater proportion of middle-aged and older individuals, facing the risk of early mortality. The *H. littoralis* population is also in decline, with few seedlings and a severe risk of local extinction. MaxEnt model predictions indicated that temperature is the primary environmental factor, with Area Under the Curve (AUC) values for all species exceeding 0.8, indicating strong predictive ability. The predicted potential suitable areas showed an expanded distribution range compared to current distribution points, providing valuable references for species introduction and propagation. **Conclusions**: This study described the population structure of the four mangrove species on the Leizhou Peninsula and assessed their primary stress factors. The results provided a theoretical basis for the conservation and restoration of endangered mangrove species and offer important guidance for developing effective conservation strategies in southern China.

## 1. Introduction

The mangroves are unique ecosystems primarily found in tropical and subtropical coastal regions, including bays, estuaries, and intertidal zones [1], and provide critical ecosystem services such as carbon sequestration, biodiversity support, and coastal protection [2]. They play a crucial role in environmental maintenance and water purification [3,4]. However, with the acceleration of urbanization and the impacts of climate change, mangroves worldwide are facing unprecedented threats [5]. Among the world’s 70 true mangrove species, 11 are classified as critically endangered or vulnerable [6]. China’s mangroves form a significant part of the global mangrove ecosystem, accounting for one-third of the world’s mangrove area. In China, the degradation of mangrove communities has led to 50% of native and semi-mangrove species becoming endangered [7]. The conservation and restoration of endangered mangroves have become focal points of societal concern and research [8,9,10]. Current studies indicate that the endangerment of mangrove species results from a combination of internal and external factors. Research into plant species, including studies on functional compounds and ecological interactions [11], is critical for understanding their conservation needs and potential applications. The documentation of newly recorded plant species is necessary not only for academic purposes but also for practical conservation efforts [12]. Internal factors include self-incompatibility, low reproductive rates, low seed germination rates, low genetic diversity, low gene flow, difficulty in seedling establishment, and intraspecific competition. External factors encompass land reclamation, estuarine hydraulic engineering, environmental pollution, climate change, overexploitation, inadequate awareness and management measures, invasive species, and interspecific competition [13,14,15]. These factors may lead to the disappearance of individual mangrove species, which could have profound impacts on the entire ecosystem [16].

The Leizhou Peninsula harbours over 9000 hectares of mangroves, accounting for 33% of China’s mangrove area and 78% of Guangdong Province’s mangrove coverage, making it the largest and most concentrated region of mangroves in China [17]. However, due to excessive development and human interference, the mangrove ecosystems of the Leizhou Peninsula are facing severe threats [18]. A thorough understanding of the status and dynamics of endangered mangrove species across the Leizhou Peninsula is crucial for formulating and implementing effective conservation and restoration strategies. Currently, there are four rare mangrove species on the Leizhou Peninsula that are considered endangered in a regional context (e.g., in China’s or Guangdong Province’s conservation lists): *Lumnitzera racemosa* Willd. (globally assessed as Least Concern (LC) by IUCN in 2018), *Ceriops tagal* (Perr.) C. B. Rob. (globally assessed as LC by IUCN in 2013), *Barringtonia racemosa* (L.) Spreng. (globally assessed as LC by IUCN in 2020), and *Heritiera littoralis* Dryand. (globally assessed as Vulnerable (VU) by IUCN in its most recent assessment). Due to the declining population of *L. racemosa*, it has been listed as an endangered species in Guangdong Province. To effectively protect the native populations of four endangered mangrove species on the Leizhou Peninsula, we need a thorough understanding of their botanical fstatus [19]. However, research on these wild populations has been neither systematic nor detailed. This lack of comprehensive study could result in the wild populations of these four endangered mangrove species being left unmanaged and excluded from effective conservation efforts. By employing population ecology methods, we can gain detailed insights into the population distribution, dynamic changes, and structural characteristics of four endangered species at different growth stages on the Leizhou Peninsula. This approach can reveal the historical structure and disturbances affecting the populations, as well as the complex relationships between the populations and their habitats [20]. Such insights offer reliable predictions for future population trends, which are crucial for formulating scientifically sound and rational conservation and management strategies [21,22,23]. Additionally, through quantitative analysis tools such as assessing age structure, life tables, survival functionality, and time series models, we can gain a deeper understanding of the current status of wild populations of *L. racemosa* on the Leizhou Peninsula, southern China. These assessments will further reveal their populational stability, ecological characteristics, and regeneration mechanisms [24,25].

## 2. Results

### 2.1. Population Distribution and Habitat Characteristics of Four Endangered Mangrove Trees in Leizhou Peninsula

**Figure 1:**
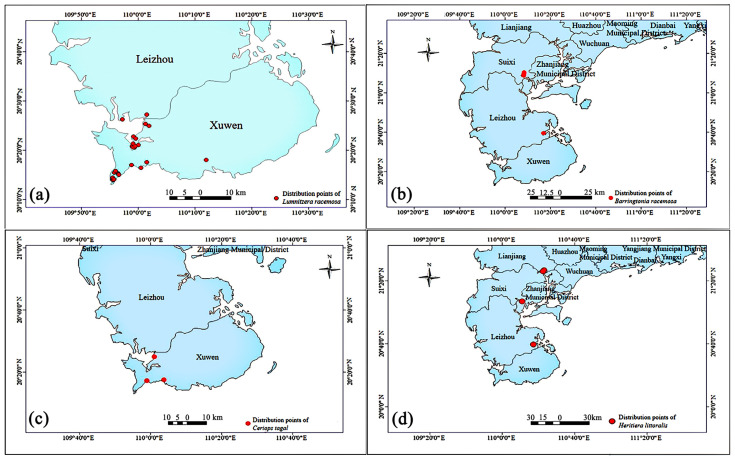
Distribution points of four endangered mangrove species on the Leizhou Peninsula. The map uses the WGS84 coordinate system. (**a**–**d**) denote the distribution of *Lumnitzera racemosa*, *Ceriops tagal*, *Barringtonia racemosa*, and *Heritiera littoralis*, respectively.

*L. racemosa* exhibits a scattered distribution across the Leizhou Peninsula, whereas *C. tagal*, *B. racemosa*, and *H. littoralis* are more concentrated (Figure 1a–d). The distribution of *L. racemosa* is primarily focused in areas such as Jiawei Township, Maichen Town, and Xilian Town in Xuwen County, covering an area of approximately 15 hectares. The mature trees can grow up to nearly 6 m in height, with a maximum basal diameter of 19.5 cm. The population is either distributed in clusters or scattered along tidal creek edges, abandoned aquaculture ponds, and the high-tide zones surrounding salt evaporation ponds (Figures S1–S3). The *L. racemosa* population exhibits high species aggregation and low diversity at the plot scale. Canopy closure is dense, though some individuals show poor growth (Tables S1 and S2, Figures S1–S3). The complexity of *L. racemosa*’s habitat and its associated plant species highlights the intricate ecological environment in which it thrives. A survey of the population’s habitat and surrounding areas recorded a total of 31 families, 45 genera, and 48 species of higher vascular plants (Table S3).

*C. tagal* is found exclusively in Maichen Town, Xuwen County, occupying an area of approximately 6100 m^2^. It forms a monospecific stand or a mixed community with Excoecaria and Sesbania, characterized by high population density and a compact structure. The individuals, with an average height of 2.2 m and an average basal diameter of 7.7 cm, exhibit a tree growth form. This stunted stature is characteristic of mangrove forests under specific environmental constraints [26], and *C. tagal* is taxonomically defined as a tree species [27]. The canopy closure is 85.4%, with approximately 1000 mature individuals in the community. The understory encompasses a dense aggregation of well-developed seedlings, with a density of 10–30 individuals/m^2^ (Tables S1 and S2, Figure S4). In the habitat and surrounding areas of the *C. tagal* population, a total of 13 families, 19 genera, and 19 species of higher vascular plants were recorded (Table S3).

*B. racemosa* is naturally distributed on both banks of the Chengyue River in Suixi County and in the Jiulongshan National Wetland Park in Leizhou City, with the community covering an area of approximately 20,000 m^2^. The trees are relatively old, with the largest basal diameter reaching over 30 cm. The average height of the *B. racemosa* population is approximately 4 m, with an average basal diameter exceeding 14 cm. The canopy closure is relatively high, ranging from 81.6% to 85% (Tables S1 and S2, Figures S5 and S6). A survey of the population’s habitat recorded a total of 34 families, 51 genera, and 52 species of higher vascular plants (Table S3), including 2 families, 2 genera, and 2 species of ferns and gymnosperms, as well as 25 families and 39 genera of angiosperms.

*H. littoralis* is found exclusively in the Jiulongshan National Wetland Park in Leizhou City, near Wenli Li Village in Mazhang District, and in Potou Village and Jilong Mountain in Lianjiang City, with approximately 216 individuals. *H. littoralis* is extremely rare and is found in the high-tide zones, where it is sparsely distributed and mixed with other plants. No seedlings of *H. littoralis* have been observed in the understory (Figures S7–S10). The average height of the *H. littoralis* population is over 4 m, with an average chest diameter exceeding 19.4 cm. The populations in Jiulongshan and Jilong Mountain are relatively taller (Tables S1 and S2). A survey of the habitat of *H. littoralis* recorded a total of 44 families, 75 genera, and 83 species of higher vascular plants (Table S3).

### 2.2. Four Endangered Mangrove Species Trees Population Height Classification

**Figure 2:**
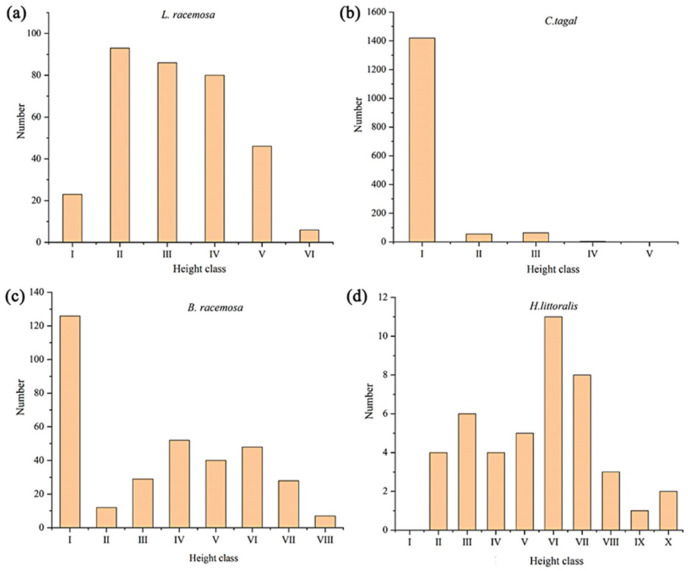
Height level structure of the four endangered mangrove species tree population. (**a**) *Lumnitzera racemosa*; (**b**) *Ceriops tagal*; (**c**) *Barringtonia racemosa*; (**d**) *Heritiera littoralis*.

The height structure analysis depicted in Figure 2a indicates that the height of all wild *L. racemosas* is below 6.0 m. Level VI has the fewest trees (only 6), accounting for 1.80% of the total, while Levels II (93 trees) and III (86 trees) have the most, accounting for 27.8% and 25.7% of the total, respectively (Figure 2a). The height of the *C. tagal* population is below 4.0 m, with the majority of individuals in height class I, followed by class III (64 individuals), and the fewest in class IV (3 individuals). This height distribution indicates a large number of seedlings within the *C. tagal* population (Figure 2b). The height of the *B. racemosa* population is below 8.0 m, with the highest number of individuals in height class I (126 individuals), and the fewest in height class VIII (7 individuals). These classes account for 36.8% and 2.0% of the total population, respectively. The height distribution shows a bimodal pattern, with a relatively abundant number of seedlings (Figure 2c). The height of *H. littoralis* individuals is below 10 m, with the largest number in height class VI (11 individuals), accounting for 25% of the total population. There are no individuals in height class I, indicating that the *H. littoralis* population is relatively small and lacks seedlings (Figure 2d).

### 2.3. Age Structure of the Four Endangered Mangrove Species Trees Population

**Figure 3:**
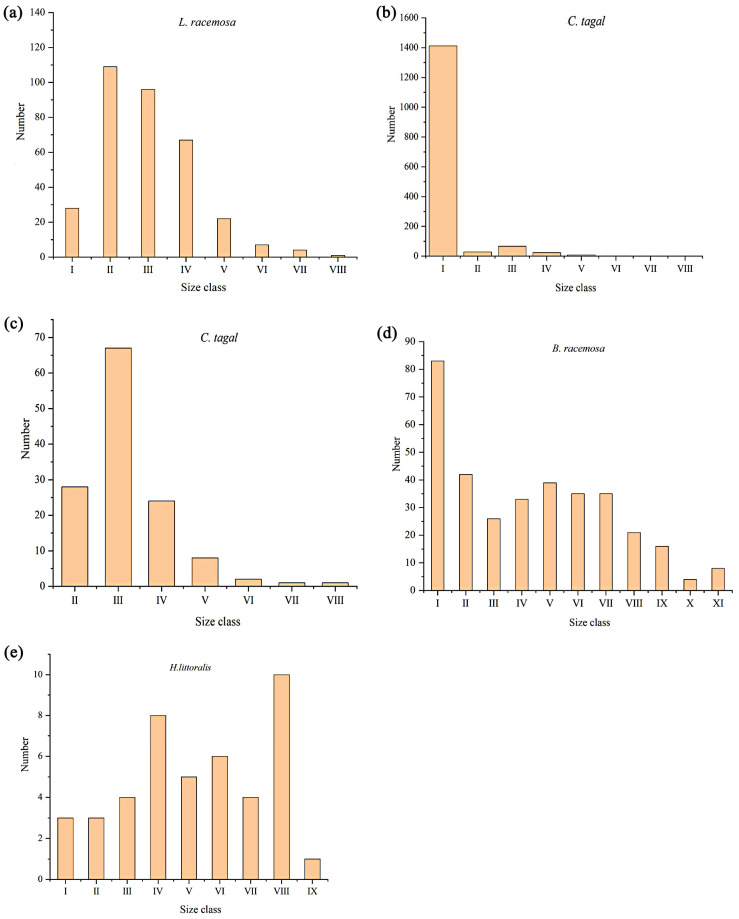
Age class structure of the four endangered mangrove species tree population. (**a**–**e**) denote the population height classification of *Lumnitzera racemosa*, *Ceriops tagal* (**b**,**c**), *Barringtonia racemosa*, and *Heritiera littoralis*, respectively.

The space-for-time substitution method was used, with the diameter class structure of *L. racemosa* replacing the age class structure. Individuals in age class I account for 8.4% of the total population. The total population gradually decreases with increasing age class beyond age class II, with the highest numbers of individuals in age classes II and III, at 109 and 96 individuals, respectively, accounting for 61.4% of the total population. Individuals in age classes IV to V make up 26.6%, while those in age classes VI to VIII account for 3.6%. A noticeable decrease in numbers occurs during the transition between age classes IV and V (Figure 3a). The number of *C. tagal* seedlings is high (1411 individuals), constituting 91.50% of the total population. To offer a clearer understanding of the population structure of *C. tagal,* excluding age class I, Figure 3b shows the age structure after excluding class I. It is evident that, after removing class I, age class III has the largest number of individuals (67 individuals), accounting for 4.35% of the total population. Age class II has 28 individuals (1.82% of the total), and individuals in age classes IV to VIII are fewer, together comprising 2.33% of the total (Figure 3c). In *B. racemosa*, the largest number of individuals is found in age class I (83 individuals), making up 24.27% of the total survey sample. This is followed by age class II with 39 individuals (11.40%), age classes III to V with 98 individuals (28.65%), and age classes VI to VIII with 91 individuals (26.61%). Individuals in age classes IX to XI total 28 individuals, accounting for 8.19% (Figure 3d). The number of young *H. littoralis* individuals in age class I is the smallest (3 individuals), accounting for 6.8%, all having a chest diameter greater than 3.5 cm. Individuals in age classes II to V account for 45.5% of the total population, while those in age classes VI to IX account for 47.7% (Figure 3e).

### 2.4. Static Life Table of the Four Endangered Mangrove Species Trees Population

For the population of *L. racemosa* on the Leizhou Peninsula, the standardized survival number ($l_{x}$) at age class I is relatively low, increases in age classes I–II, and then decreases with increasing age from age class II to VIII. There is a sharp decline in standardized survival numbers between age classes III–V. The corresponding values in the life table, including standardized mortality ($d_{x}$), mortality rate ($q_{x}$), cumulative mortality rate ($F_{x}$), and disappearance rate ($K_{x}$), show negative values (Table S4). Life expectancy decreases initially in age classes I–IV, then increases in age classes IV–VI, and decreases again in age classes VI–VIII, with the lowest mean survival ability observed at age class VIII (0.50).

For the population of *C. tagal*, the standardized survival number at age class II is lower than that at age class III, resulting in negative values for standardized mortality ($d_{x}$), mortality rate ($q_{x}$), and disappearance rate ($K_{x}$) in the life table (Table S4). Life expectancy ($e_{x}$) fluctuates across age classes, with the highest value observed at age class II (4.18). Life expectancy decreases between age classes III–IV and then increases again at age classes VI and VII, surpassing the levels of age classes III–V.

For *B. racemosa*, the individual standard survival number decreases from age classes I to III, increases from age classes III to V, and decreases again from age classes V to X. This leads to negative values in the life table for standard mortality ($d_{x}$), mortality rate ($q_{x}$), and disappearance rate ($K_{x}$) (Table S4). The population’s life expectancy ($e_{x}$) shows an increasing trend from age classes I to III, peaking at age class III (7.85), with a general trend of increasing life expectancy that later decreases as age class increases.

The standard survival number of *H. littoralis* fluctuates across different age classes, indicating a highly unstable population. In age classes II, III, V, and VII, the standard survival number of *H. littoralis* is lower than that of the following age class, resulting in negative values for standard mortality ($d_{x}$), mortality rate ($q_{x}$), and disappearance rate ($K_{x}$). The cumulative mortality rate ($F_{x}$) remains negative from age classes II to VII (Table S4). The life expectancy ($e_{x}$) of *H. littoralis* is highest in age class I (14.17), decreasing from age classes I to IV. Afterward, life expectancy shows a relatively stable fluctuation trend, but a significant decline occurs in age classes VIII and IX.

### 2.5. Survival Curve, Mortality Rate Curve, and Disappearance Rate Curve of the Four Endangered Mangrove Species Trees Population

**Figure 4:**
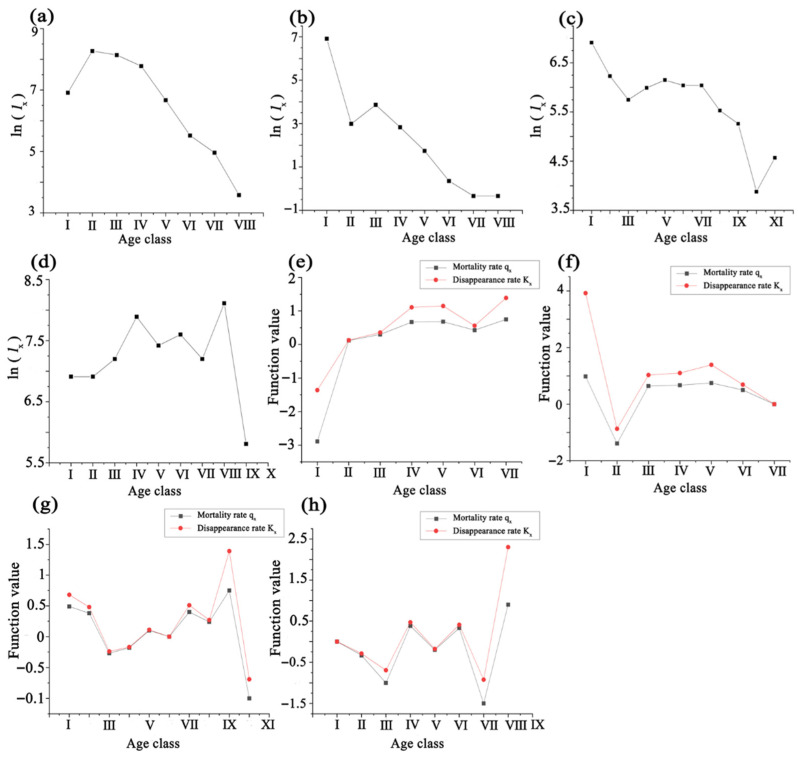
The survival curve, mortality, and disappearance rate curves of the four endangered mangrove species tree populations. (**a**–**d**) denote the population survival curves of *Lumnitzera racemosa*, *Ceriops tagal*, *Barringtonia racemosa*, and *Heritiera littoralis*, respectively; (**e**–**h**) denote the mortality rate curves and disappearance rate curves of *Lumnitzera racemosa*, *Ceriops tagal*, *Barringtonia racemosa*, and *Heritiera littoralis*, respectively.

The survival curve of the *L. racemosa* population follows a Deevey Type II pattern. The slope of the curve is relatively shallow from age classes II to IV, after which it slightly increases (Figure 4a). As the population approaches its physiological lifespan, the number of survivors decreases. Overall, the curve remains relatively flat, with mortality stabilizing after age class II, showing minimal variation.

The mortality rate curve of the *L. racemosa* population mirrors the trend of the disappearance rate curve (Figure 4e). A significant increase is observed from age class I to II, followed by a slower rise as the population stabilizes. The mortality rate in age class V is 0.68, while the disappearance rate is 1.15. The maximum values occur in age class VII, with a mortality rate of 0.75 and a disappearance rate of 1.39. Overall, both mortality and disappearance rates exhibit a rising–falling–rising pattern.

The survival curve of the *C. tagal* population follows a concave growth pattern, intermediate between the Deevey III and Deevey II types (Figure 3b). Deevey II indicates a stable population with relatively uniform mortality across age classes, while Deevey III represents a stress-tolerant community with high seedling mortality but long-lived adults. The slope of the curve is steep from age class I to II, with a low survival rate for seedlings (age class I).

The mortality and disappearance rate curves of the *C. tagal* population show similar trends, exhibiting a pattern of decrease–increase–decrease (Figure 4f). The mortality and disappearance rates are highest in age class I ($q_{x}$ = 0.98, $K_{x}$ = −3.92), reaching their lowest values in age class II ($q_{x}$ = −1.39, $K_{x}$ = −0.87). A secondary peak in both mortality and disappearance rates occurs from age classes II to V ($q_{x}$ = 0.75, $K_{x}$ = 1.39). From age classes V to VII, both mortality and disappearance rates decrease, with the surviving *C. tagal* individuals entering their mature phase.

The population curve of *B. racemosa* follows an initial concave and then convex trend, lying between the Deevey Type I and Type III curves (Figure 4c). The number of individuals rapidly decreases between age classes I–III, with a steeper slope, indicating a low survival rate for seedlings and a challenging transition to mature trees. Starting at age class III, the curve shifts to a convex shape, and the slope of the curve gradually flattens. After age class IX, the survival rate drops sharply, signaling the physiological end of the lifespan of *B. racemosa*, with a rapid decline in population size.

After entering age class III, the *B. racemosa* population reaches a relatively stable state, with both mortality and disappearance rates following a general trend of decrease–increase–decrease (Figure 4g). The mortality and disappearance rates at age class I are relatively high ($q_{x}$ = 0.49, $K_{x}$ = 0.68), gradually decreasing until age class III. From age classes III to IX, both mortality and disappearance rates fluctuate and increase, reaching their peak at age class IX ($q_{x}$ = 0.75, $K_{x}$ = 1.39). However, between age classes IX and X, there is a sharp decline in both rates.

The survival curve of the *H. littoralis* population follows a Deevey Type I pattern (convex curve), with a low proportion of juveniles and a higher proportion of middle-aged and older individuals. The standardized survival logarithm of seedlings is low, with a slight increase observed from age classes I to IV. Afterward, it fluctuates and decreases between age classes IV and VII, rises at age class VIII, and then rapidly decreases (Figure 4d).

The mortality and disappearance rates of the *H. littoralis* population exhibit a general pattern of decrease–increase–decrease–increase–decrease–increase (Figure 4h). Mortality and disappearance rates for individuals in age classes I–III show a slight decrease, followed by a fluctuating increase from age classes III to VI. A sharp decline occurs at age class VI, reaching the lowest values ($q_{x}$ = −1.50, $K_{x}$ = −0.92). At age class VIII, both mortality and disappearance rates peak. During this stage, *H. littoralis* faces difficulties transitioning to age class IX.

### 2.6. Survival Analysis of the Four Endangered Mangrove Species Tree Population

**Figure 5:**
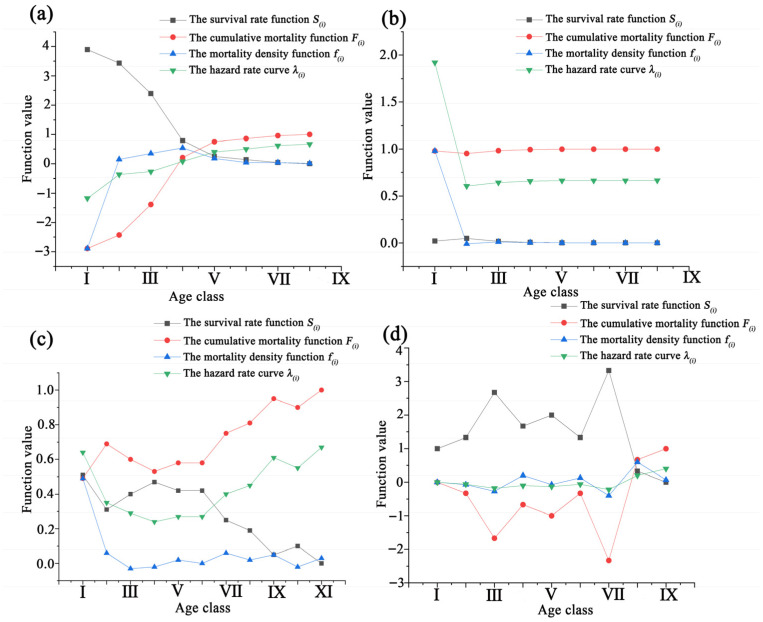
The survival rate function $S_{\left(i\right)}$, the cumulative death function $F_{\left(i\right)}$, the mortality density function $f_{\left(i\right)}$, and the hazard rate curve $\lambda_{\left(i\right)}$ for the four endangered mangrove species tree population. (**a**–**d**) denote the survival rate function, the cumulative death function, the mortality density function, and the hazard rate curve for *Lumnitzera racemosa*, *Ceriops tagal*, *Barringtonia racemosa*, and *Heritiera littoralis*, respectively.

As shown in Figure 5a, the survival function of the *L. racemosa* population exceeds the cumulative mortality function from age classes I to V. At age classes V–VI, the two functions intersect and reach equilibrium, after which the survival function falls below the cumulative mortality function, indicating the onset of population decline. The survival function gradually decreases with increasing age, while the cumulative mortality rate steadily increases. The fluctuations of both functions are most pronounced in age classes I–IV, after which they level off. Starting at age class VII, the cumulative mortality rate exceeds 96%, marking the beginning of the physiological decline phase. The mortality density function differs somewhat from the hazard rate curve, which illustrates variations in instantaneous mortality risk across different age classes and provides insights into population stability and the effects of environmental stressors. The mortality density function rises sharply after age class I, with the lowest mortality density observed at age class I ($f_{\left(i\right)}$ = −2.89). It increases through age classes II–IV, reaching its maximum at age class IV ($f_{\left(i\right)}$ = 0.54). In contrast, the hazard rate curve of *L. racemosa* increases steadily, peaking at age class VIII ($\lambda_{\left(i\right)}$ = 0.67), indicating higher risks for the population in the later stages of life.

**Figure 6:**
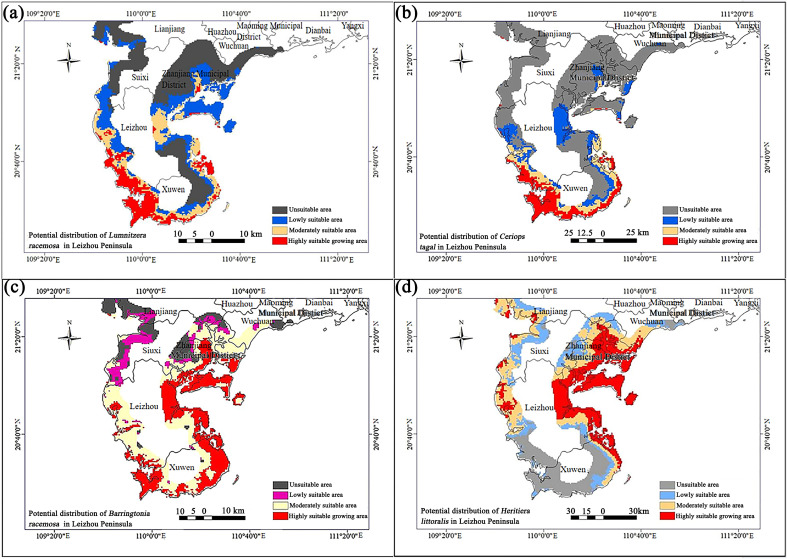
Potential distribution of the four endangered mangrove species tree populations on the Leizhou Peninsula. (**a**–**d**) denote the potential distribution of *Lumnitzera racemosa*, *Ceriops tagal*, *Barringtonia racemosa*, and *Heritiera littoralis* on the Leizhou Peninsula, respectively.

As shown in Figure 6b, the survival function of the *C. tagal* population remains lower than the cumulative mortality function across age classes I to VIII. The population survival rate begins to decline from age class II, while the cumulative mortality rate steadily increases. Throughout the age classes, the survival rate is consistently lower than the hazard rate. The mortality density function of the *C. tagal* population differs from the hazard rate curve, exhibiting fluctuations. The mortality density is highest in age class I ($f_{\left(i\right)}$ = 0.9802), decreases at age class II, increases at age class III, and then continues to decline thereafter. The hazard rate curve shows that the hazard rate peaks in age class I ($\lambda_{\left(i\right)}$ = 1.92), lowest at age class II ($\lambda_{\left(i\right)}$ = 0.6062), and then gradually increases at a small rate thereafter.

As shown in Figure 5c, the survival function of *B. racemosa* exceeds the cumulative mortality function at age class I, but it falls below the cumulative mortality function afterward, indicating the onset of population decline. The survival function decreases initially, then increases, and finally decreases again with increasing age, while the cumulative mortality rate rises, then falls, and rises again. The fluctuations in both functions are more pronounced in the later stages than in the earlier stages, with the two functions being complementary but never intersecting. The cumulative mortality rate exceeds 90% at age class IX, signaling the entry of individuals into the physiological decline phase.

The mortality density function of the *B. racemosa* population differs from the hazard rate curve. The mortality density decreases rapidly after age class I, where mortality density is highest ($f_{\left(i\right)}$ = 0.49), and the hazard rate is also relatively high ($\lambda_{\left(i\right)}$ = 0.64). The mortality density remains relatively stable between age classes II and XI, while the hazard rate curve decreases from age classes I to IV, reaching its lowest point at age class IV ($\lambda_{\left(i\right)}$ = 0.24). Afterward, the hazard rate gradually increases, peaking at age class XI ($\lambda_{\left(i\right)}$ = 0.67).

As shown in Figure 5d, the survival function exceeds the cumulative mortality function from age classes I to VIII, with an intersection occurring between age classes VII and VIII, where the two functions reach equilibrium and complement each other. The survival function increases with age in a fluctuating pattern, with small peaks at age classes III and VII, the latter reaching its maximum value ($S_{\left(i\right)}$ = 3.33). In contrast, the cumulative mortality rate follows a fluctuating decreasing trend, with two troughs at age classes III and VII, the latter reaching its minimum value ($F_{\left(i\right)}$ = −2.33). The mortality density function of *H. littoralis* shows a degree of fluctuation, peaking at age class VIII ($f_{\left(i\right)}$ = 0.60), while the hazard rate curve peaks at age class IX ($\lambda_{\left(i\right)}$ = 0.40).

### 2.7. Quantitative Dynamic Analysis of the Four Endangered Mangrove Species Trees Population

Based on population dynamic quantification methods, the changes in the individual numbers between adjacent age classes of the *L. racemosa* population on the Leizhou Peninsula were analyzed. The dynamic results of the population numbers between age classes (V_n_) are as follows: −74.31%, 11.93%, 30.21%, 67.16%, 68.18%, 42.86%, and 75%, indicating a “decline–growth–growth–growth–growth–growth–growth” structure (Table S5). In the absence of external disturbances, the population dynamic index (*V_pi_*) is 26.18%. When external disturbances are considered, the population dynamic index (*V′_pi_*) drops to 3.273%, with the maximum probability of random disturbance risk (*P_max_*) reaching 0.125. Since *V_pi_* is greater than *V′_pi_*, and both values are greater than 0, the overall population is classified as growth-oriented.

The population dynamic results for *C. tagal* across age classes show the following values for *V_n_*: 98.04%, −58.21%, 64.18%, 66.67%, 75.00%, 50.00%, and 0.00%, indicating a “growth–decline–growth–growth–growth–growth–stability” structure (Table S5). The population dynamic index (*V_pi_*) is 92.99%, while the adjusted index (V′_pi_) is 11.624%, with the maximum probability of random disturbance risk (*P_max_*) at 0.125. Therefore, the overall population is classified as growth-oriented.

The population dynamic results for *B. racemosa* between age classes show the following values for *V_n_*: 49.40%, 38.10%, −21.21%, −15.38%, 10.26%, 0.00%, 40.00%, 23.81%, 75.00%, and −50%, indicating a “growth–growth–decline–decline–growth–stability–growth–growth–growth–decline” structure (Table S5). The population dynamic index (*V_pi_*) is 23.77%, while the adjusted index (*V′_pi_*) is 0.547%, with the maximum probability of random disturbance risk (*P_max_*) at 0.023. Therefore, the overall population is classified as growth-oriented.

The population dynamic results for *H. littoralis* between age classes show the following values for *V_n_*: 0.00%, −25%, −50%, 37.50%, −16.70%, 33.30%, −60.00%, and 90%, indicating a “stability–decline–decline–growth–decline–growth–decline–growth” structural relationship, with the population’s development being significantly hindered (Table S5). The population dynamic index (*V_pi_*) is 18.63%, while the adjusted index (*V′_pi_*) is 2.068%, with the maximum probability of random disturbance risk (*P_max_*) at 0.111. Although *V_pi_* is greater than *V′_pi_*, both values are relatively small, with the highest contribution occurring in the older age classes (V_8_). Considering the survival function, the mortality risk is high for older plants, indicating that the *H. littoralis* population is overall in a decline phase.

### 2.8. Time Series Analysis of the Four Endangered Mangrove Species Tree Populations

The population time series analysis shows a high level of predictive accuracy. The single moving average method from time series analysis was used to predict the dynamics of four populations. The prediction results (Table S6) indicate that after two age classes, the population of *L. racemosa* experiences a decline in the number of individuals in age class II, while the remaining age classes show an increase. After four, six, and eight age classes, every age class continues to increase, with the peak number shifting towards larger diameter classes.

In the absence of large-scale mortality in age class I seedlings, the population of *C. tagal* was predicted using the single moving average method based on the original number of individuals across all age classes. The prediction results (Table S6) indicate that after two, four, six, and eight age classes, the number of individuals increases across all age classes. After age class II, the greatest increase is observed in age class II (with an increase of over 24 times). After four age classes, the greatest increase is observed in age class IV; after six age classes, age class VI exhibits the largest increase, with the peak number shifting towards larger diameter classes.

For the *B. racemosa* population, the prediction results (Table S6) indicate that after two age classes, the number of individuals in age classes IV, V, and XI decreases, while the remaining age classes increase. Age class II shows the greatest increase (48.8%), which is aligns with the population dynamic analysis. After four age classes, the number of individuals in age classes V and VI decreases, while the remaining age classes increase. After six and eight age classes, the number of individuals increases in all age classes except for age class VII, which shows no variation.

For the *H. littoralis* population, the prediction results (Table S6) indicate that after two age classes, the number of individuals in age classes III, IV, V, and VIII decreases. After four, six, and eight age classes, the number of individuals decreases in most age classes, while the number of individuals in age class IX increases. This indicates that the *H. littoralis* population on the Leizhou Peninsula faces severe extinction risks.

### 2.9. Prediction of the Potential Distribution of Four Endangered Mangrove Species Trees

Using the Potential Distribution Prediction Method, the distribution data (Table S7) of the four endangered mangrove species and environmental variables were imported into MaxEnt for pre-modeling. After 10 simulation runs, environmental factors with very low contribution rates were excluded, and iterative second-round modeling was conducted. The second-round modeling produced the ROC curve and AUC values (Figure 1). For *L. racemosa*, the average AUC value from ten runs was 0.900, with the best simulation achieving an AUC value of 0.948. The average AUC for *C. tagal* was 0.941, with the best simulation achieving an AUC value of 0.978. The average AUC for *B. racemosa* was 0.814, with the best training AUC value at 0.927 and the testing AUC at 0.976. The average AUC for *H. littoralis* was 0.852, with the best training AUC value at 0.912 and the testing AUC at 0.940. All ten simulation runs produced AUC values between 0.8 and 1.0, all significantly higher than the random probability value, indicating that the simulations were successful, and the model can differentiate known locations from background points, making it suitable for predicting the potential suitable distribution areas of these four endangered wild mangrove species.

The contribution of each environmental variable to the prediction model (Table S8) is outlined as follows: for *L. racemosa*, Wettest Quarter Mean Temperature (32.3%), Coldest Quarter Mean Temperature (17.2%), Warmest Quarter Mean Temperature (12.4%), Annual Mean Temperature (11.3%), Mean Diurnal Range (6.1%), and Average Sea Surface Temperature of Coldest Month (4.3%) totaled 83.1%. For *C. tagal*, Wettest Quarter Mean Temperature (32.8%), Minimum Temperature of Coldest Month (24.6%), Annual Mean Temperature (20.2%), Mean Diurnal Range (4.8%), Maximum Temperature of Warmest Month (4.0%), and Mean Sea Surface Temperature (3.3%) totaled 89.8%.

For *B. racemosa*, the contribution percentages of Annual Mean Temperature (37.8%), Wettest Quarter Mean Temperature (35.4%), Precipitation of Driest Month (7.0%), Precipitation of Wettest Month (4.2%), Mean Diurnal Range (3.9%), and Mean Temperature of Warmest Quarter (3.7%) totaled 92%. For *H. littoralis*, Wettest Quarter Mean Temperature (46.5%), Mean Diurnal Range (31.1%), and Precipitation of Driest Month (11.4%) accounted for a total of 89%.

The response curves of the primary contributing environmental factors to the prediction model (Figures S19–S21) indicate that the distribution probability of *L. racemosa* is higher within the following environmental parameters: Wettest Quarter Mean Temperature between 28.6 and 31.8 °C, Coldest Quarter Mean Temperature between 16.7 and 21.9 °C, Warmest Quarter Mean Temperature between 28.5 and 29.4 °C, Annual Mean Temperature between 23.5 and 25.8 °C, Mean Diurnal Range between 5.5 and 6.8 °C, and Average Sea Surface Temperature of Coldest Month between 15 and 22.5 °C. For *C. tagal*, the distribution probability is higher within the following parameters: Wettest Quarter Mean Temperature around 28.0–31.6 °C, Minimum Temperature of Coldest Month around 14.0–22.5 °C, Annual Mean Temperature around 23.8–26 °C, Mean Diurnal Range around 3.5–7.3 °C, Maximum Temperature of Warmest Month between 32.3 and 33 °C, and Mean Sea Surface Temperature between 23.3 and 27.6 °C. For *B. racemosa*, the distribution probability is higher within the following parameters: Annual Mean Temperature between 22.7 and 28.1 °C, Wettest Quarter Mean Temperature between 27.5 and 31.6 °C, Precipitation of Driest Month between 0 and 47 mm, Precipitation of Wettest Month between 100 and 391 mm, Mean Diurnal Range between 3.5 and 7.3 °C, and the Mean Temperature of Warmest Quarter between 28.1 and 31.7 °C. For *H. littoralis*, the distribution probability is higher within the following parameters: Wettest Quarter Mean Temperature between 27.6 and 31.8 °C, Mean Diurnal Range between 3.3 and 6.7 °C, Precipitation of Driest Month between 23 and 48 mm, temperature seasonal variability standard deviation between 420 and 590 °C, and Precipitation of Coldest Quarter between 90 and 141 mm.

The species presence probability maps of the four endangered wild mangrove species, obtained from the MaxEnt model, were imported into ArcGIS Pro 2.9 and converted into raster data. Reclassification was performed using the natural breaks classification method to delineate the potential suitable distribution areas for the species. The potential suitable distribution area for *L. racemosa* is primarily located in the southern part of the Leizhou Peninsula, including suitable habitats in Xuwen County and Leizhou City, such as Qindou Town, Xilian Town, Maichen Town, Yingli Town, Liusha Bay, Xinliao Town, and Dongsonghai (Figure 6a). The potential distribution area of *C. tagal* overlaps with that of *L. racemosa* to some extent but is situated further south, with its northern boundary lower than that of *L. racemosa*. It is primarily distributed in a belt-like pattern across the southern part of the Leizhou Peninsula, including Xilian Town, Maichen Town, Liusha Bay, Qianshan Town, Xinliao Town, and Duzigang, where theoretical potential planting areas for *C. tagal* are located (Figure 6b).

The potential distribution range of *B. racemosa* is relatively wide, with high-suitability areas in Suixi County, Leizhou City, and Xuwen. These areas are primarily concentrated on the eastern and southern sides of the Leizhou Peninsula, including Tongminghai, Leizhou Port, Dongsonghai, Chengyue River, Jinhe Town, Xinliao Town, He’an Town, Tiaofeng Town, Taiping Town, and Jianxin Town, where there are theoretical potential planting areas for *B. racemosa* (Figure 6c). The potential suitable distribution area for *H. littoralis* is primarily located on the eastern side of the Leizhou Peninsula. High-suitability areas are found in Lianjiang City, Suixi County, Leizhou City, and Xuwen, specifically including Tongminghai, Leizhou Port, Dongsonghai, Jinhe Town, Xinliao Town, He’an Town, Tiaofeng Town, Taiping Town, Jianxin Town, Guandu Town, Liangdong Town, and Suixi River. These regions encompass theoretical potential planting areas for *H. littoralis* (Figure 6d).

## 3. Discussion

### 3.1. Population Dynamics of Four Endangered Mangrove Species Across the Leizhou Peninsula

Plant populations undergo various stages from germination to migration, including growth, competition, and environmental adaptation, ultimately establishing stable ecological niches. The population’s age and height structure provide key insights into its successional trends and regeneration potential [28]. Population dynamics can be analyzed through methods such as static life tables, survival curves, and quantitative dynamics analysis to assess environmental adaptation and regeneration capacity, which are critical for the conservation of endangered species [29,30].

This study shows that the populations of *L. racemosa*, *B. racemosa*, *C. tagal*, and *H. littoralis* across the Leizhou Peninsula exhibit distinct differences in structure and dynamics. The *H. littoralis* population follows a decline pattern with an “inverted pyramid” age structure, dominated by middle-aged and old individuals, and a lack of juveniles. This pattern is similar to that of *Firmiana pulcherrima* in Hainan [31], reflecting severe habitat degradation. In contrast, the *H. littoralis* population in Baguang, Shenzhen, benefits from a healthy habitat and a balanced age structure [32]. The discrepancy suggests that the Leizhou Peninsula population faces greater environmental stress, hindering regeneration.

Populations of *L. racemosa*, *B. racemosa*, and *C. tagal* show a “pyramid” structure, suggesting growth potential. However, *L. racemosa* exhibits an atypical structure, with very few seedlings compared to juvenile trees, indicating low germination rates. This limitation parallels the situation of *Clematis acerifolia* in the Taihang Mountains [33]. The scarcity of seedlings hinders the population’s growth and regeneration capacity. Conversely, *C. tagal* and *B. racemosa* populations have abundant seedlings, but both face high mortality during the seedling-to-juvenile transition, limiting population renewal. This pattern is also observed in *Michelia wilsonii* and *Sonneratia ovata* [34]. While these species show growth potential, their seedling replenishment is unstable, raising concerns about long-term regeneration.

Life expectancy ($e_{x}$) reflects survival ability and environmental adaptation. Both *L. racemosa* and *H. littoralis* exhibit high life expectancy in seedlings, but low survival rates due to strong environmental selection. This aligns with the findings of Jin et al. [35] on *Rhododendron chrysanthum* and Yang et al. [36] on *Rhododendron rex*. *C. tagal* and *B. racemosa* show higher life expectancy after transitioning to juvenile trees, indicating improved adaptability at later stages, consistent with Luo et al. [37] on *Hopea hainanensis*.

The survival curves and mortality rates offer additional insights into population trends. *L. racemosa* follows a Deevey II curve, while *C. tagal* and *B. racemosa* show early mortality but stabilize later. *H. littoralis* follows a Deevey I curve, with fluctuating survival rates, indicating population instability. These patterns suggest that different life stages face unique survival challenges, consistent with studies on other endangered species [38,39]. These results highlight the complexity of survival dynamics in endangered species.

Quantitative analysis and time series analysis reveal the overall developmental trends of these populations. *L. racemosa* shows a decline at the seedling stage but an overall growth trend, while *C. tagal* and *B. racemosa* exhibit a decline in the juvenile stage but still show overall growth. *H. littoralis* experiences decline across multiple age classes, reflecting poor population replenishment [40,41]. Population dynamics are shaped by habitat conditions, succession processes, and human interference [30]. Time series analysis indicates that, under favorable conditions, the populations of *L. racemosa*, *C. tagal*, and *B. racemosa* will grow, while *H. littoralis* will primarily see an increase in older individuals, with inadequate replenishment of younger cohorts. This analysis underscores the importance of human-assisted conservation measures to stabilize populations and promote sustainable growth [42,43].

### 3.2. Potential Habitat Suitability and Endangerment Causes of Four Endangered Mangrove Species in the Leizhou Peninsula

The MaxEnt model has proven effective in predicting the spatial distribution of endangered species and assessing their responses to environmental changes, including those affecting mangrove ecosystems [44,45,46,47,48,49]. In the present study, MaxEnt simulations yielded AUC values exceeding 0.900 for all four endangered mangrove species in the Leizhou Peninsula, indicating strong model performance. These results are consistent with Ying et al. [50], who reported a high correspondence between predicted high-suitability areas and actual distribution of *Kandelia obovata* (S.,L.) Yong, underscoring the model’s reliability.

Among the primary environmental drivers, temperature-related bioclimatic variables were the most influential across all four species, aligning with previous research on mangrove distribution in Xiamen Bay and Guangdong Province [14]. However, the dominant predictors and their contributions varied among species, reflecting differences in ecological requirements and environmental sensitivities [51,52]. Notably, current distribution points generally coincide with high-suitability areas, suggesting that model predictions can guide conservation planning, including site selection for species introduction and habitat restoration.

For most species, high-suitability zones were broader than current distribution ranges. An exception was *B. racemosa*, which had occurrence points outside predicted optimal areas, potentially indicating localized microclimatic conditions that facilitate its survival. Overall, the model outputs offer a scientifically sound basis for identifying priority areas for in situ and ex situ conservation. Future work should incorporate additional environmental and anthropogenic variables, as well as climate projections, to refine predictions and support adaptive management strategies.

Despite their differing ecological niches, the four species—*H. littoralis*, *C. tagal*, *B. racemosa*, and *L. racemosa*—share similar threats. Among them, *H. littoralis* is the most critically endangered, characterized by a severely diminished population and almost no seedling recruitment, indicating a loss of self-regeneration capacity. *L. racemosa* maintains relatively more juvenile individuals despite low seedling numbers, suggesting some reproductive potential. *C. tagal* and *B. racemosa* show active seedling emergence but experience bottlenecks during the transition to juvenile stages, compromising long-term population viability.

Anthropogenic disturbances are the primary drivers of mangrove loss. Rapid economic development has led to widespread mangrove habitat conversion for aquaculture, salt production, agriculture, and urban expansion. Additional pressures, such as sewage discharge and coastal infrastructure (e.g., cement roads), have further degraded these habitats [53]. Habitat fragmentation reduces genetic diversity and limits population resilience [54], exacerbating the vulnerability of already small and isolated populations.

Moreover, biological invasions by fast-growing vines such as *Mikania micrantha* and *Ipomoea pes-caprae* pose additional threats, particularly to *B. racemosa* and *H. littoralis*. These invasive species compete aggressively for space and nutrients, suppressing mangrove seedling development and potentially leading to localized dieback.

Effective conservation strategies must address both intrinsic biological limitations and extrinsic environmental pressures. Priorities should include improving seed germination and seedling survival, enhancing asexual propagation, and restoring degraded habitats to support natural regeneration. Model-informed site selection, combined with in situ protection and off-site conservation of critically impacted populations, can optimize resource allocation. Management interventions, such as clearing dead biomass in overly dense stands to increase light penetration, may further support population recovery.

### 3.3. Future Research Perspectives

Building upon this study, several key directions emerge for future research to deepen our understanding and improve the conservation of these endangered mangrove species. First, future work should move beyond using correlative distribution models to incorporate mechanistic ecophysiological studies. Quantifying the specific physiological thresholds and adaptive capacities of these species to stressors like salinity fluctuation, inundation, and low temperature will elucidate the underlying drivers of their distribution limits and mortality bottlenecks, particularly for vulnerable seedling stages. Second, to enhance the predictive accuracy for conservation planning, it is crucial to integrate future climate change scenarios into species distribution models. Projecting the shifts in suitable habitats listed under different IPCC pathways will help to identify areas that may become future climate refugia or facing potential heightened risk, enabling proactive and climate-resilient conservation strategies. Finally, integrating relevant molecular tools with ecological field studies represents a transformative multi-disciplinary frontier. Population genomic analyses can assess the genetic diversity and inbreeding levels of these small, isolated populations, identify functional adaptations, and inform assisted gene flow strategies. Furthermore, exploring the composition and function of associated soil and root microbiomes could reveal novel pathways to enhance seedling growth and eco-physiological stress resilience, offering innovative approaches for ecological restoration. By coupling advanced modeling, physiological insights, and genetic tools, future research can progress from describing population decline to actively reversing it through scientifically grounded, multifaceted interventions.

## 4. Materials and Methods

### 4.1. Overview of the Study Area

#### 4.1.1. Geographic Location

**Figure 7:**
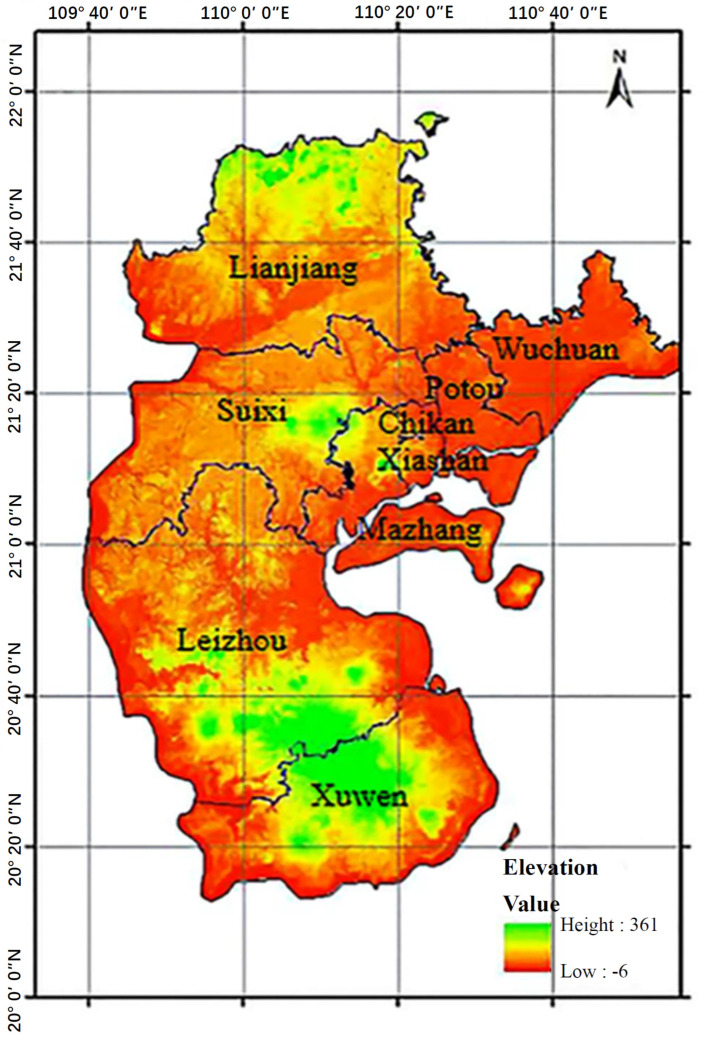
Location and elevation map of the Leizhou Peninsula, Guangdong Province, China. The map uses the WGS84 coordinate system.

Located at the southernmost tip of the Chinese mainland (109°31′–110°55′ E, 20°12′–21°35′ N), the Leizhou Peninsula is bordered by the South China Sea to the east, the Beibu Gulf to the west, and separated from Hainan Island by the Qiongzhou Strait to the south. The peninsula spans approximately 140 km in length and 60–70 km in width, covering an area of about 7800 km^2^. The geographic location and area roughly correspond to those of the current jurisdiction of Zhanjiang City, making it a crucial juncture on the Maritime Silk Road (Figure 7). Its terrain is characterized by gentle, low-lying platforms and basalt terraces, with the highest elevations found in the northern regions. The peninsula experiences a typical South Asian tropical monsoon climate, with a mean annual temperature of 23 °C and annual precipitation ranging from 1400 to 1700 mm. The coastline extends approximately 1180 km, or 1450 km including islands, and hosts diverse mangrove ecosystems. Surveys have recorded 8 families and 13 species of true mangroves, accounting for 50% of China’s total true mangrove species, along with 8 families and 10 species of semi-mangroves, representing 83.33% of the national total. Dominant species include *Kandelia obovata* (S., L.) Yong., *Avicennia marina* (Forsk.) Vierh., and *Rhizophora stylosa* Griff. The mangrove communities generally exhibit a shrub-like structure, with canopy heights typically between 2 and 4 m. A clear zonation pattern exists along the tidal gradient: pioneer species such as *Avicennia marina* dominating the seaward margins, followed by *K. obovata* and *R. stylosa* species in the intermediate zones, and more complex, semi-terrestrial communities further inland.

#### 4.1.2. Overview of Mangrove Resources

Through an assessment of regional species richness and endangered species, the Leizhou Peninsula has been identified as one of the biodiversity hotspots for mangrove communities in China. The distribution of mangroves on the Leizhou Peninsula extends from Gaoqiao Town in Lianjiang City in the west, to Wuli in Nanshan Town, Xuwen County in the south (20°15′ N), and to Wuyang Town, Wuchuan City in the east (21°30′ N), covering the muddy beaches [53]. The Zhanjiang Mangrove National Nature Reserve in Guangdong Province, located on the Leizhou Peninsula, is the largest, most diverse, and most concentrated coastal mangrove reserve in China. The reserve is rich in biodiversity, with 16 families and 25 species of true mangroves and semi-mangroves, 48 families and 311 species of birds, 58 families and 127 species of fish, 13 families and 37 species of crustaceans, and 68 families and 147 species of benthic animals [55].

In this study, the semi-mangroves refer to a category of woody plants that possess characteristics of both mangroves and terrestrial plants [6,48]. They typically inhabit the margins of mangrove forests, the upper tidal zones, or coastal terrestrial areas affected by salt spray [26]. While they lack the highly specialized morphological structures of true mangroves (such as vivipary and aerial roots), they can tolerate certain levels of soil salinity and intermittent seawater immersion [27]. These species are considered transitional communities between mangrove and terrestrial ecosystems [56]. Common semi-mangrove species in the study region include *Cerbera manghas* L., *Hibiscus tiliaceus* (L.) Fryxell, and *Pongamia pinnata* (L.) Pierre [48,57].

### 4.2. Field Investigation

#### 4.2.1. Investigation Method

In the first stage, we reviewed literature and specimen collection data to understand the historical distribution of four endangered mangrove species [8,9,10,17,53]. We consulted with authors and experts to grasp the current research status on the Leizhou Peninsula, identified potential distribution areas, and conducted a field survey to assess the distribution, population characteristics, and threat levels of four endangered mangrove species.

In the second stage, we conducted a detailed survey of crucial mangrove areas on the Leizhou Peninsula. We established typical sample plots (10 m × 10 m or 5 m × 5 m) in areas with concentrated distributions of the four endangered mangrove species, following a modified phytosociological approach centered on a complete population census [58]. Within each plot, we conducted a full census, recording the number, height, breast diameter, basal diameter, and crown width of all individuals with a basal diameter ≥1 cm.

For seedlings with a basal or breast diameter <1 cm, only the species name and height were recorded. We also documented other plant species (identified via ground-based survey), geographical coordinates, altitude, and beach type for each plot. Additionally, drone aerial images were acquired to provide complementary data on canopy structure and to assist in accurately georeferencing and mapping the plot boundaries.

For areas with sparse and dispersed populations of four endangered mangrove species, we used the transect (belt) method and the full investigation method. The transect method was suitable for larger, more dispersed populations, while the full investigation method was used for smaller, more localized populations.

#### 4.2.2. Tools and General Equipment

Measurement tools: tape measures, calipers, roll-up rulers, tree height meters, soil pH conductivity meters, Drone, GPS devices, etc.

Recording tools: survey forms, survey maps, recording pens, notebooks, cameras, work bags, etc. Specimen collection tools: collection bags, specimen clips, branch shears, iron shovels, ropes.

### 4.3. Population Dynamics Analysis Methods

#### 4.3.1. Height Class Analysis

Drawing on the methods used by Liang et al. [13]. For classifying the height classes of mangrove forests, this study divides the height structure of general mangrove plants into classes of 1.0 m increments, such as Class I: 0.0–1.0 m, Class II: 1.0–2.0 m, Class III: 2.0–3.0 m, and so forth, with specific classes set according to the final measurement data.

#### 4.3.2. Age Structure Analysis

Most ecologists prefer to use diameter classes as a surrogate for age classes in studies of the population dynamics and structure of endangered species [10,59]. In this study, the size classes for wild *L. racemosa* trees are adapted from Ning et al. [60] and Hu et al. [41] for mangrove community grading. Plants with a basal diameter (DBH) <1 cm are classified as Class I. For plants with a DBH ≥1 cm, each 3 cm increase in DBH corresponds to a new class, with Class II: 1 cm ≤ DBH < 4cm, Class III: 4 cm ≤ DBH < 7 cm, Class IV: 7 cm ≤ DBH < 10cm, Class V: 10 cm ≤ DBH < 13 cm, Class VI: 13 cm ≤ DBH < 16 cm, and so on.

#### 4.3.3. Life Table Compilation

Age structure is crucial for understanding population dynamics. This study uses a diameter-class structure to analyze the populations of four endangered mangrove species. By substituting space for time, we create a static life table based on age classes [61].

Although survey errors may cause negative mortality rates, many scholars opt not to standardize data to better reflect ecological processes. Therefore, we refer to the data processing methods of Hu et al. [41], Tian et al. [55], and Lv et al. [62]. The calculation formulas for various indices in the life table are as follows:

(1) $$l_{x}=\frac{a_{x}}{a_{1}}\times 1000,$$

(2) $$d_{x}=l_{x}-l_{x+1},$$

(3) $$q_{x}=\frac{d_{x}}{l_{x}},$$

(4) $$L_{x}=\frac{l_{x}+l_{x+1}}{2},$$

(5) $$T_{x}=\sum_{x}^{\infty }L_{x},$$

(6) $$e_{x}=\frac{T_{x}}{l_{x}},$$

(7) $$S_{x}=\frac{l_{x+1}}{l_{x}},$$

(8) $$F_{x}=\frac{\sum_{1}^{x}d_{x}}{l_{x}},$$

(9) $$K_{x}=\ln l_{x}-\ln l_{x+1},$$

In Formulas (1)–(9), $a_{x}$ reflects the existing number of survivors in age class x (individuals); $l_{x}$ reflects the standard number of survivors at the beginning of age class x (individuals); $d_{x}$ reflects the standard number of deaths between age class x and x + 1 (individuals); $q_{x}$ reflects the period mortality rate between age class x and x + 1; $L_{x}$ reflects the standard number of surviving individuals between age class x and x + 1 (individuals); $T_{x}$ reflects the total number of standard individuals in age class x and above (individuals); $e_{x}$ reflects the average life expectancy of individual plants in age class x; $S_{x}$ reflects the survival rate of the population; $F_{x}$ reflects the cumulative mortality rate; and $K_{x}$ reflects the extinction rate.

#### 4.3.4. Construction of the Survival Curves, Mortality Curves, and Extinction Rate Curves

Based on the life table, the age classes of four endangered mangrove species are used as the horizontal axis, and the natural logarithm of the number of survivors ($ln\;l_{x}$), the interval mortality rate ($q_{x}$), and the extinction rate ($K_{x}$) as the vertical axes to plot the survival curve, interval mortality rate curve, and extinction rate curve of the four endangered mangrove species tree populations.

#### 4.3.5. Survival Analysis

To better analyze the population structure of four endangered mangrove species trees and clarify their survival patterns, this study incorporates four functions from survival analysis into the population survival analysis. These include the population survival function $S_{\left(i\right)}$, cumulative mortality function $F_{\left(i\right)}$, mortality density function $f_{\left(i\right)}$, and hazard rate function $\lambda_{\left(i\right)}$. The calculation formulas for these functions are as follows:

(10) $$S_{\left(i\right)}=S_{1}\times S_{2}\times S_{3}\times \cdots \times S_{i},$$

(11) $$F_{i}=1-S_{\left(i\right)},$$

(12) $$f_{\left(i\right)}=(S_{\left(i-1\right)}-S_{\left(i\right)})/h_{i},$$

(13) $$\lambda_{\left(i\right)}=2(1-S_{\left(i\right)})/[h_{i}(1+S_{\left(i\right)})],$$

In the formulas, $S_{i}$ reflects the survival frequency, $h_{i}$ reflects the age class width. Based on these functions, we plot the survival rate curve, cumulative mortality rate curve, mortality density curve, and hazard rate curve.

#### 4.3.6. Analysis of Quantitative Indicators for Population Dynamics

This study refers to the method proposed by Chen et al. [29] for quantitatively analyzing plant population dynamics. The formulas for each dynamic index are as follows:

(14) $$V_{n}=\frac{S_{n}-S_{n+1}}{max(S_{n},S_{n+1})},$$

(15) $$V_{pi}=\frac{\sum_{n=1}^{K-1}\left(S_{n}{\times V}_{n}\right)}{\sum_{n=1}^{K-1}S_{n}},$$

(16) $$P_{max}=\frac{1}{K\times min(S_{1},S_{2},S_{3},\cdots ,S_{k})},$$

(17) $$V_{pi}^{\prime }=V_{pi}\times P_{max},$$

In the formulas, $V_{n}$ reflects the dynamic index of individual number changes in the population from age class n to n + 1, reflecting the relationship between the number of individuals in adjacent age classes (across the entire age structure of the population). A positive value indicates growth, a negative value indicates decline, and zero indicates stability. $V_{pi}$ reflects the dynamic index of number changes for the entire population structure. $S_{n}$ and $S_{n+1}$ reflect the number of individuals in the nth and (n + 1) th age classes, respectively. K reflects the maximum age class in the population. $P_{max}$ reflects the maximum probability of the entire population bearing random disturbances. $V_{pi}^{\prime }$ is the sensitivity index of population structure dynamics to random disturbances, reflecting the dynamic index of number changes in the entire population structure when the population is subject to random disturbances.

#### 4.3.7. Time Series Analysis

We employed the simple moving average method to forecast the population dynamics of four endangered mangrove species tree populations for 2, 4, 6, and 8 years into the future. The calculation formula is as follows [63]:

(18) $$M_{t}=\frac{1}{n}\sum_{k=t-n+1}^{t}X_{k},$$

In the formula, ‘n’ reflects the time to be forecasted, ‘t’ stands for the age class, $X_{k}$ denotes the current survival number of the kth age class population, and $M_{t}$ indicates the survival number of the tth age class population after the future n years. Applying the above model, the total number of endangered mangrove plant populations and the number of plants at each diameter class surveyed serve as the original data, which are calculated using the simple moving average method.

### 4.4. Potential Distribution Prediction Method

#### 4.4.1. Acquisition and Processing of Plant Geographical Distribution Data

The geographical distribution data of plant distribution points are primarily obtained through two methods:

(1) Latitude and longitude data are recorded using GPS based on previous field survey sampling data (Table S7).
(2) Search for four endangered mangrove species’ distribution information on the Chinese Virtual Herbarium (https://www.cvh.ac.cn), the Global Biodiversity Information Facility (https://www.gbif.org/, accessed in 15 May 2025), and the China Academic Journals Full-text Database. Coordinates for those not specifically marked with latitude and longitude information are obtained through map positioning. Location information is accurate to below the county level.

In the MaxEnt model, known distributions and constraints need to be imported simultaneously in a specified format. Species coordinates are unified into decimal form in Excel and saved as a CSV file. The longitude and latitude data are imported into ArcGIS 10.6, and distribution points with autocorrelation are removed using the SDM Toolbox.

#### 4.4.2. Selection and Processing of Environmental Variable Data

We used 19 bioclimatic variables and 5 sea surface environmental factors to predict the potential distribution area of four endangered mangrove species. The bioclimatic factors for modeling come from worldclim.org; and ocean data from bio-oracle.org. For specific information, see Table S9. This study imported climatic variables and sea surface variables into the ArcGIS Pro 2.9 tool and used the extension tool to merge and connect the two. The environmental variables are unified in the ArcGIS Pro 2.9 software boundary and coordinate system (WGS-1984) and resampled to the same resolution. Finally, the marine and terrestrial environmental variables are converted into an ASC file format.

#### 4.4.3. Model Establishment

We conducted predictive analysis using MaxEnt3.4.4 software. 75% of the acquired four endangered mangrove species tree distribution data were used as training data for model establishment, while the remaining 25% were used as test data for model validation. “Create response curves” and “Make pictures of predictions” options were selected. To improve prediction accuracy, the model was set to compute repeatedly for 10 times, while other settings remained default [64]. The distribution dataset and environmental variable dataset were imported into the model for modeling. Before formal modeling, all variables were pre-modeled in Maxent, and after removing environmental factors with low contributions, secondary modeling was performed [65].

#### 4.4.4. Model Validation

The Area Under the Curve (AUC) was used to evaluate the quality of the model. When the AUC value is between 0.5 and 0.6, it indicates model failure; between 0.6 and 0.7, poor simulation effect; between 0.7 and 0.8, fair simulation effect; between 0.8 and 0.9, good simulation effect; between 0.9 and 1.0, very good simulation effect [66,67].

#### 4.4.5. Contribution Analysis

After the model operation, the relative contribution of each environment was assessed using contribution percentages. Most scholars choose to analyze the dominant environmental factors affecting plants based on their contribution [14,50].

#### 4.4.6. Environmental Response Curves

The Maxent model can generate environmental response curves. The x-axis reflects the range of environmental variable changes, while the y-axis reflects the natural logarithm of the probability of the predicted species’ existence under the corresponding environmental variable. Its value ranges from 0 to 1, with a higher value indicating a higher probability of species existence.

#### 4.4.7. Potential Suitable Distribution

The study area was ultimately set to expand by 10–20 km inland from coastal areas and estuaries [68]. The model output files were imported into ArcGIS 10.6, and the extraction tool was used to extract the results within the area range. Based on model standards and the probability of appearance of raster layers, the habitat suitability classification index range was determined, and a potential suitable distribution map of the species was drawn.

## Data Availability

Data are contained within the article and Supplementary Materials.

## Supplementary Materials

The following supporting information can be downloaded at: https://www.mdpi.com/article/10.3390/plants14213381/s1. Figure S1: Image of the population of *Lumnitzera racemosa* at location 1; Figure S2: Image of the population of *Lumnitzera racemosa* at location 2; Figure S3: Image of the population of *Lumnitzera racemosa* at location 3; Figure S4: Image of the population of *Ceriops tagal*; Figure S5: Image of the population of *Barringtonia racemosa* at location 1; Figure S6: Image of the population of *Barringtonia racemosa* at location 2; Figure S7: Image of the population of *Heritage Littoralis* at location 1; Figure S8: Image of the population of *Heritage Littoralis* at location 2; Figure S9: Image of the population of *Heritage Littoralis* at location 3; Figure S10: Image of the population of *Heritage Littoralis* at location 4; Figure S11: Validation results of ROC curve for the *Lumnitzera racemosa* prediction model (Average); Figure S12: Validation results of ROC curve for the *Lumnitzera racemosa* prediction model (Optimum); Figure S13: Validation results of ROC curve for the *Ceriops tagal* prediction model (Average); Figure S14: Validation results of ROC curve for the *Ceriops tagal* prediction model (Optimum); Figure S15: Validation results of ROC curve for the *Barringtonia racemosa* prediction model (Average); Figure S16: Validation results of ROC curve for the *Barringtonia racemosa* prediction model (Optimum); Figure S17: Validation results of ROC curve for the *Heritiera littoralis* prediction model (Average); Figure S18: Validation results of ROC curve for the *Heritiera littoralis* prediction; Figure S19: The main environmental factor response curves contributed by the *Lumnitzera racemosa* prediction model; Figure S20: The main environmental factor response curves contributed by the *Ceriops tagal* prediction model; Figure S21: The main environmental factor response curves contributed by the *Barringtonia racemosa* prediction model; Table S1: Basic overview of the mangrove sample plots in Guangdong, China; Table S2: Population characteristics of four mangrove species across different sampling sites; Table S3: List of associated plants in the habitats of four mangrove and coastal tree species; Table S4: Static life tables for mangrove species populations in Leizhou Peninsula; Table S5: Population dynamic indices (Vn, Vpi, V′pi, Pmax) across age-class transitions for four mangrove species; Table S6: Dynamic time-series table of population quantities for four mangrove species; Table S7: Consolidated geographical distribution data of four mangrove species; Table S8: Contribution percentages of environmental variables in species distribution prediction models; Table S9: Environmental variables dataset.

## Abbreviations

The following abbreviations are used in this manuscript:

AUC	Area Under the Curve
EN	Endangered
