# Supplementary material for: Population Dynamics and Potential Distribution of the Four Endangered Mangrove Species in Leizhou Peninsula China
Source: Plants (Basel). 2025 Nov 5;14(21):3381. doi: 10.3390/plants14213381 (PMC12609941; doi:10.3390/plants14213381)
Supplement: Supplementary file 1 [file plants-14-03381-s001.zip › plants-3927101-supplementary.pdf]

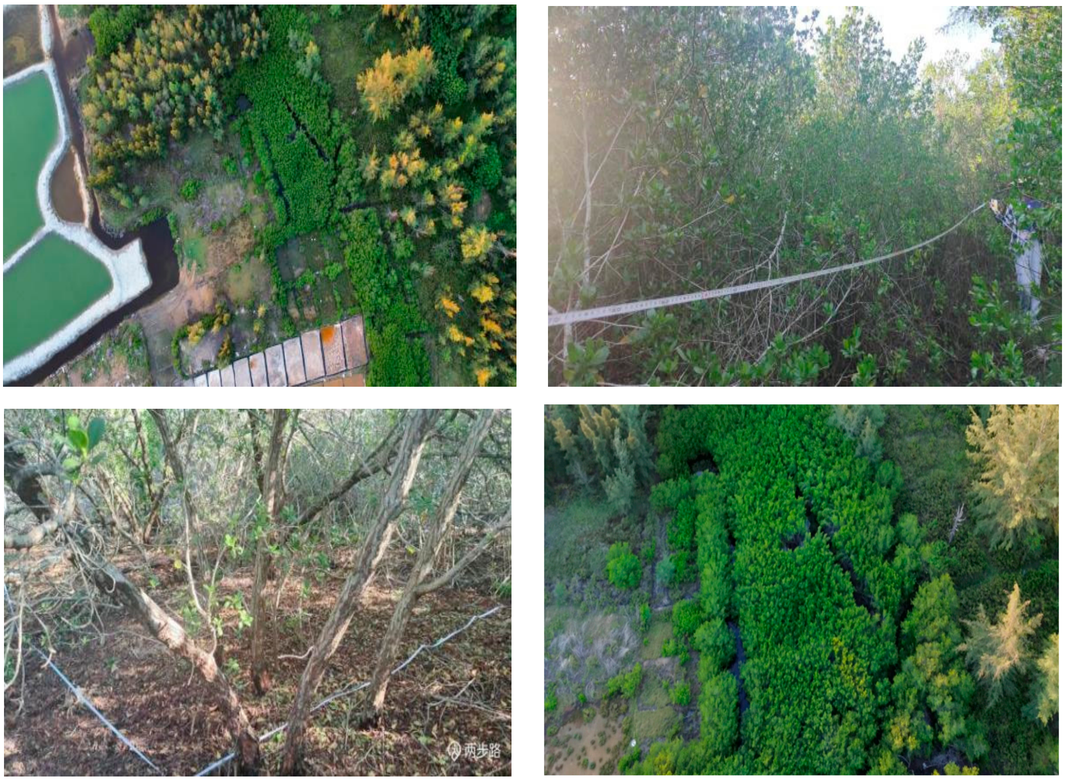

Figure S1. Image of the population of *Lumnitzera racemosa* at location 1

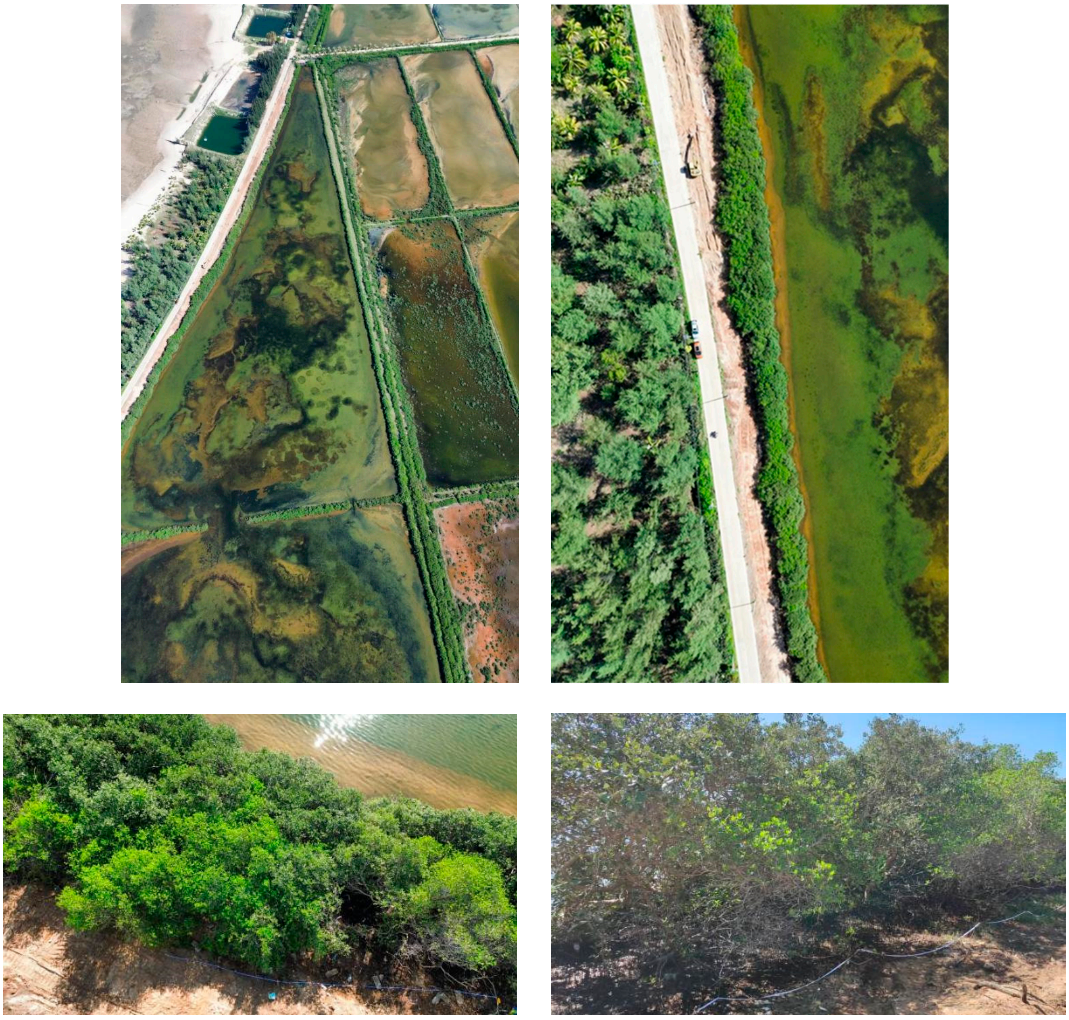

Figure S2. Image of the population of *Lumnitzera racemosa* at location 2

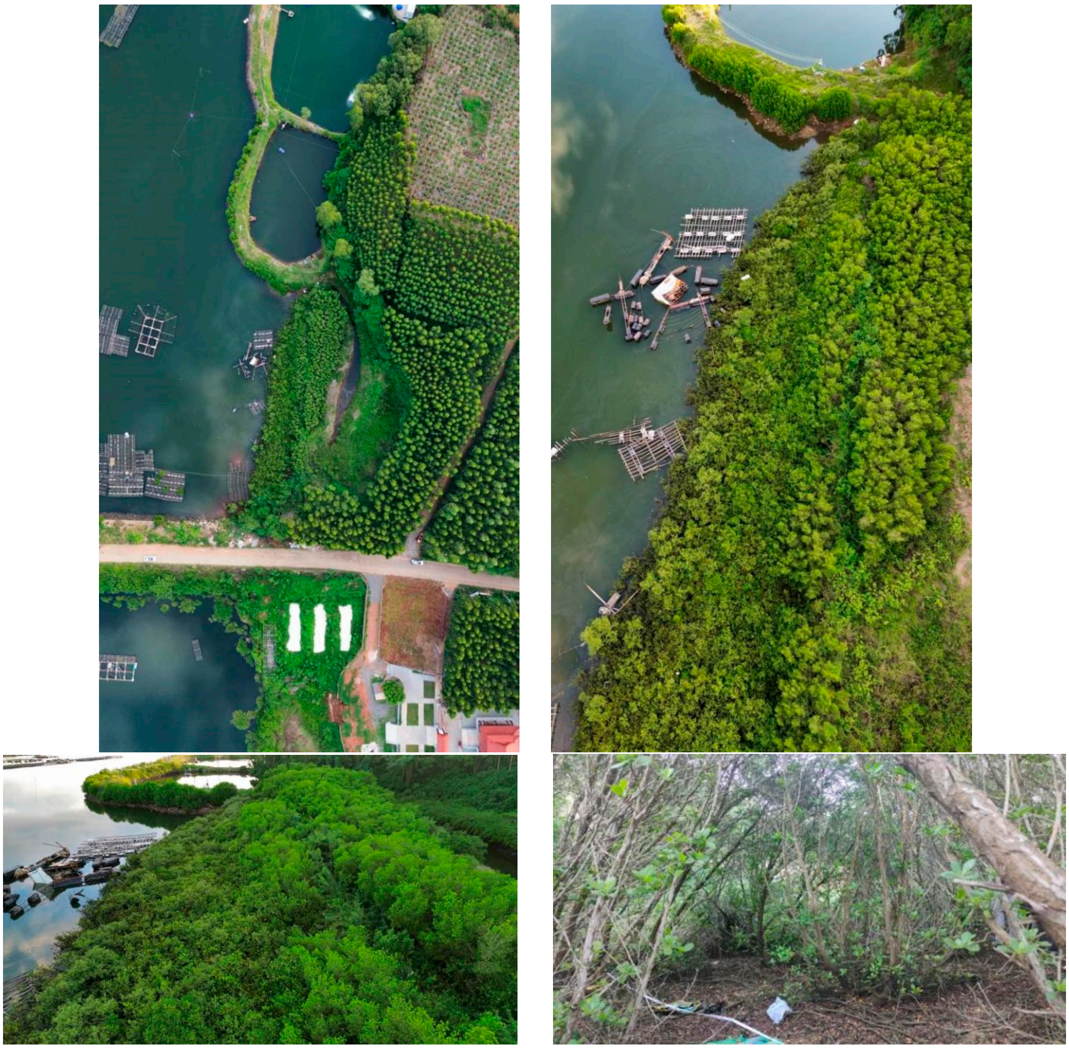

Figure S3. Image of the population of *Lumnitzera racemosa* at location 3

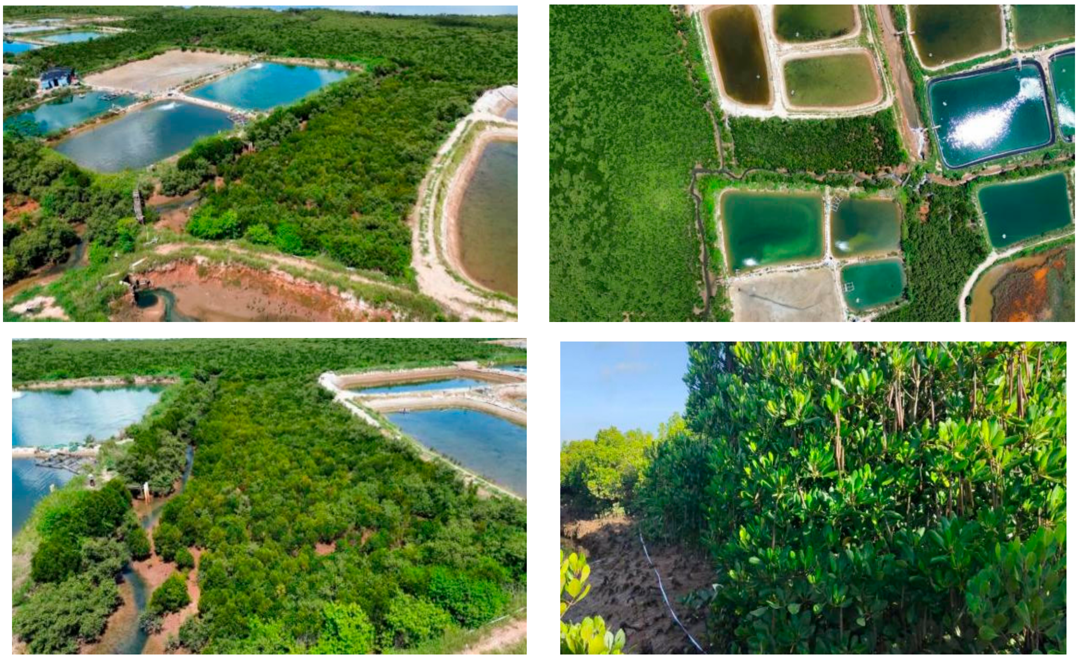

Figure S4. Image of the population of *Ceriops tagal*

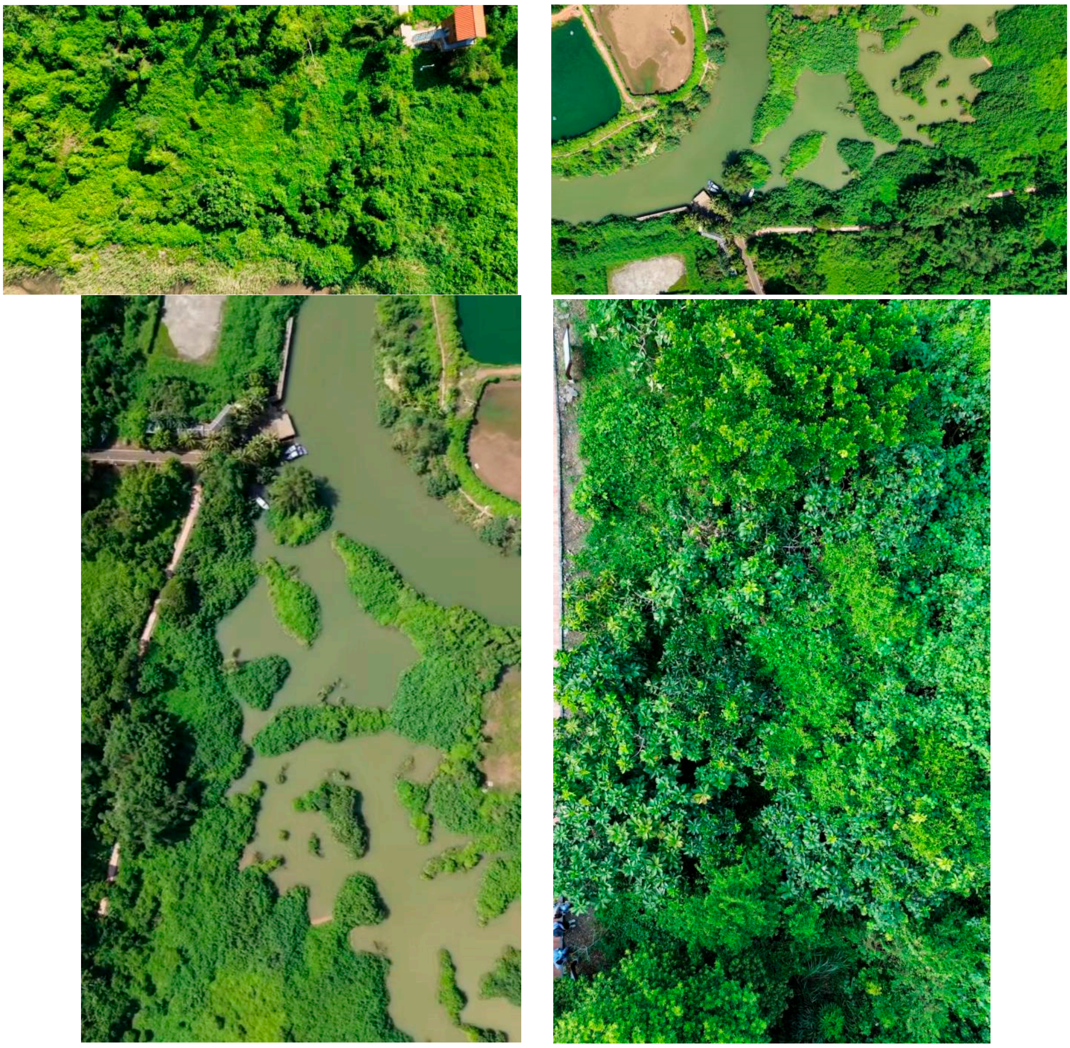

Figure S5. Image of the population of *Barringtonia racemosa* at location 1

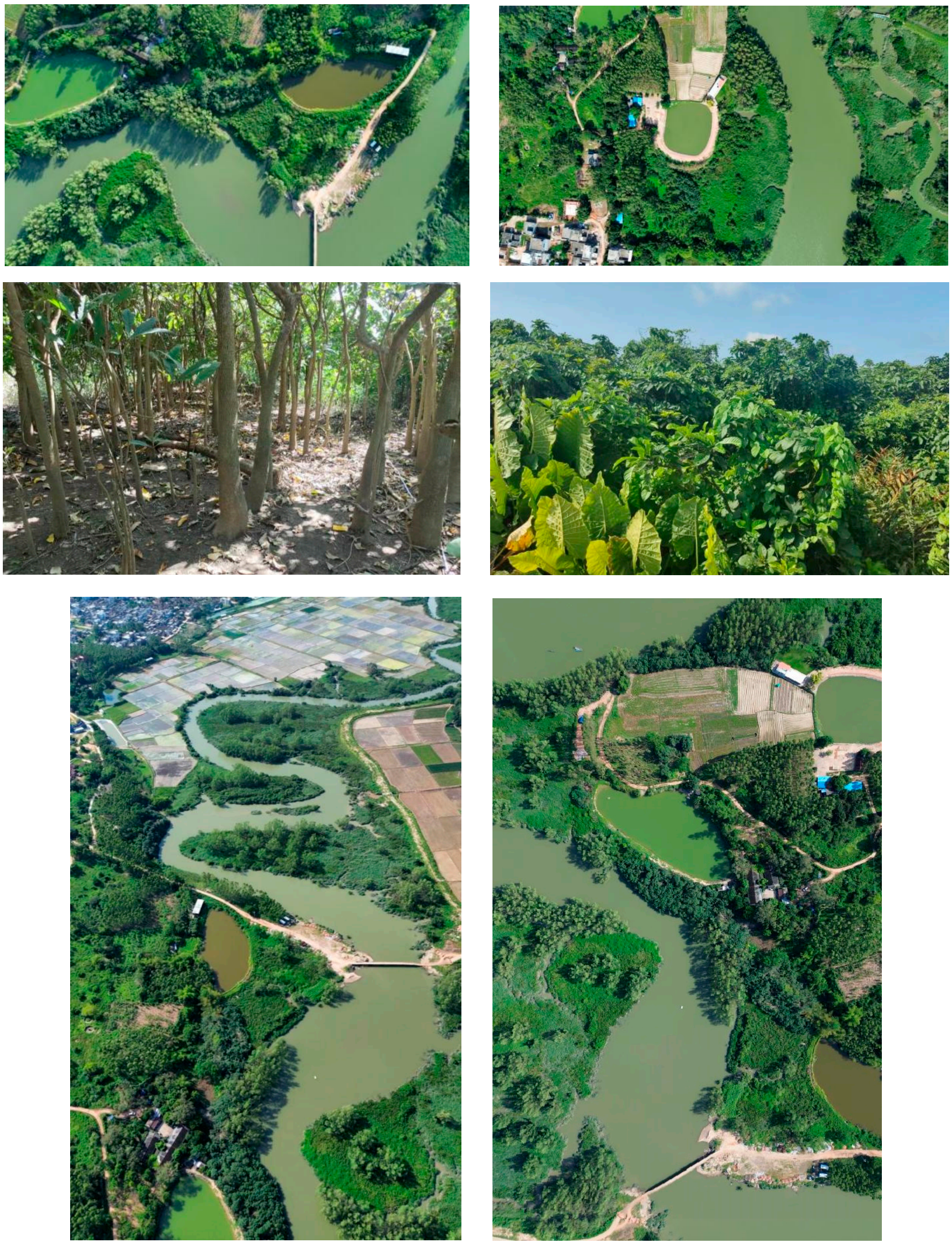

Figure S6. Image of the population of *Barringtonia racemosa* at location 2

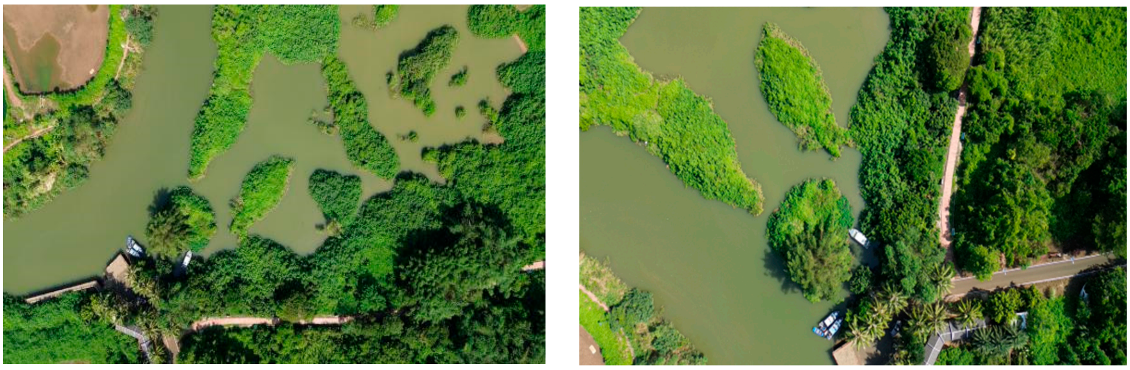

Figure S7. Image of the population of *Heritage Littoralis* at location 1

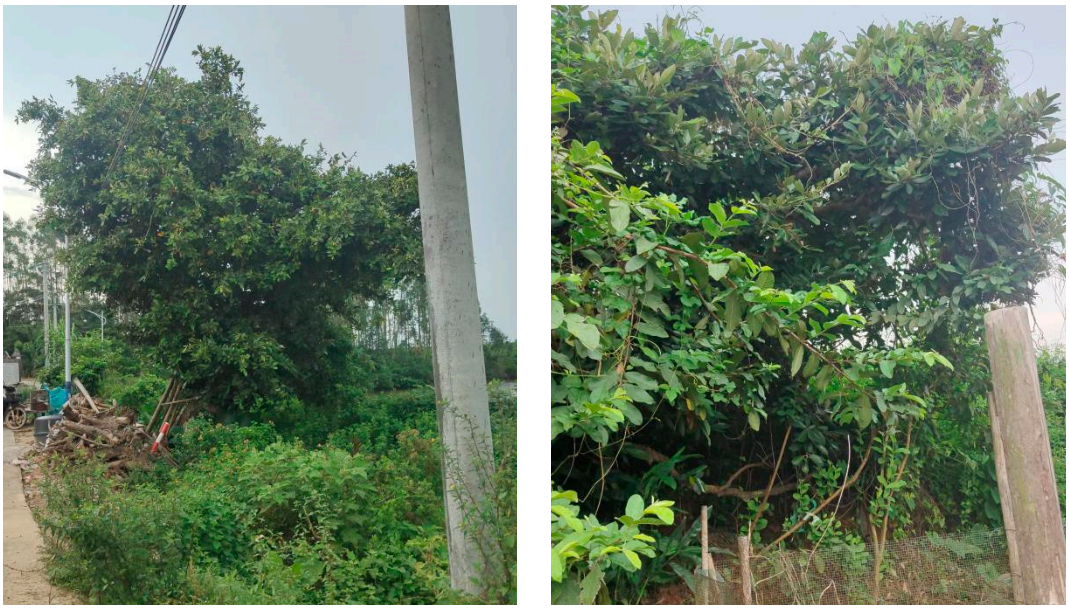

Figure S8. Image of the population of *Heritage Littoralis* at location 2

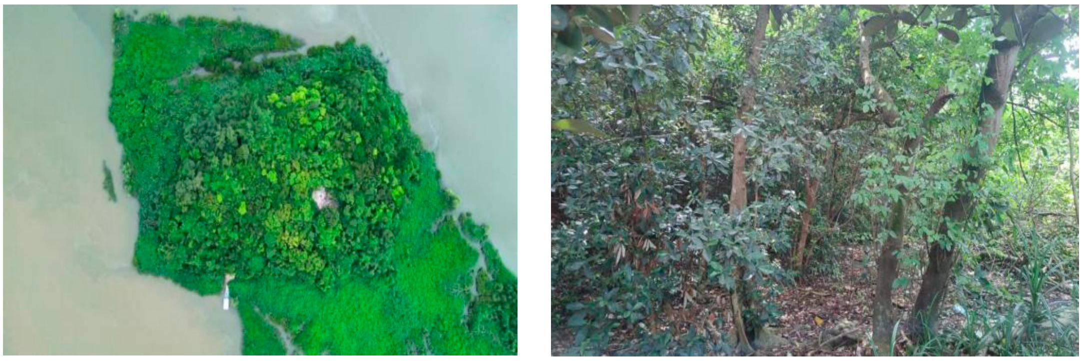

Figure S9. Image of the population of *Heritage Littoralis* at location 3

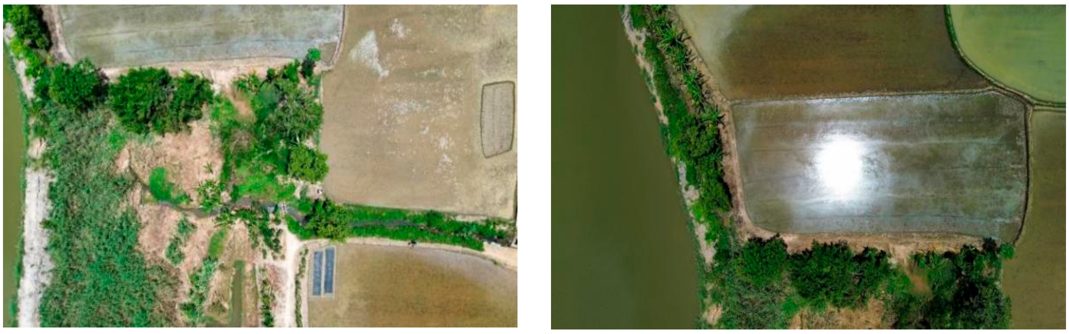

Figure S10. Image of the population of *Heritage Littoralis* at location 4

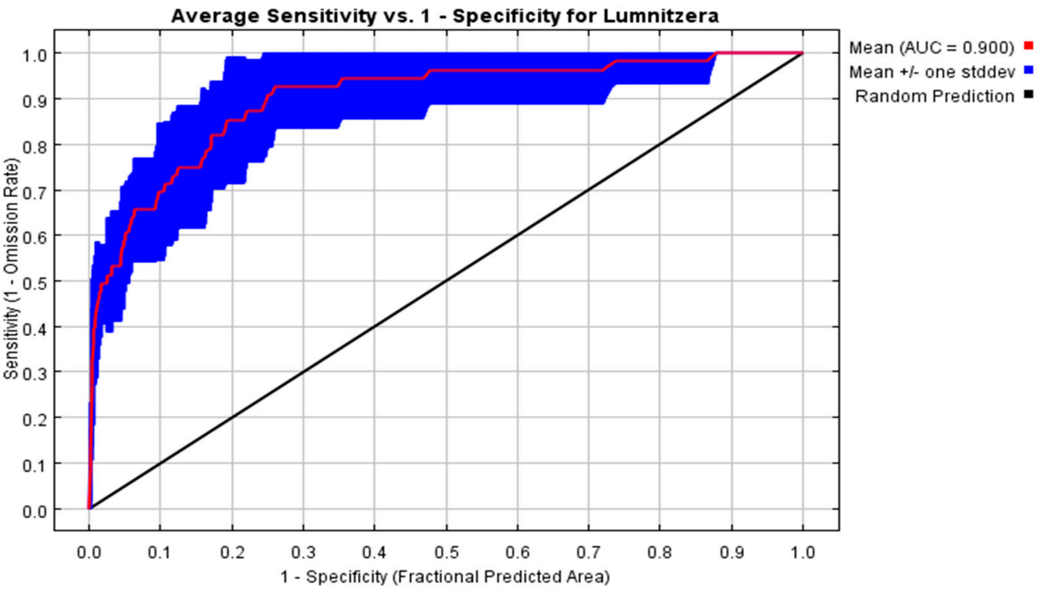

Figure S11. Validation results of ROC curve for the *Lumnitzera racemosa* prediction model (Average)

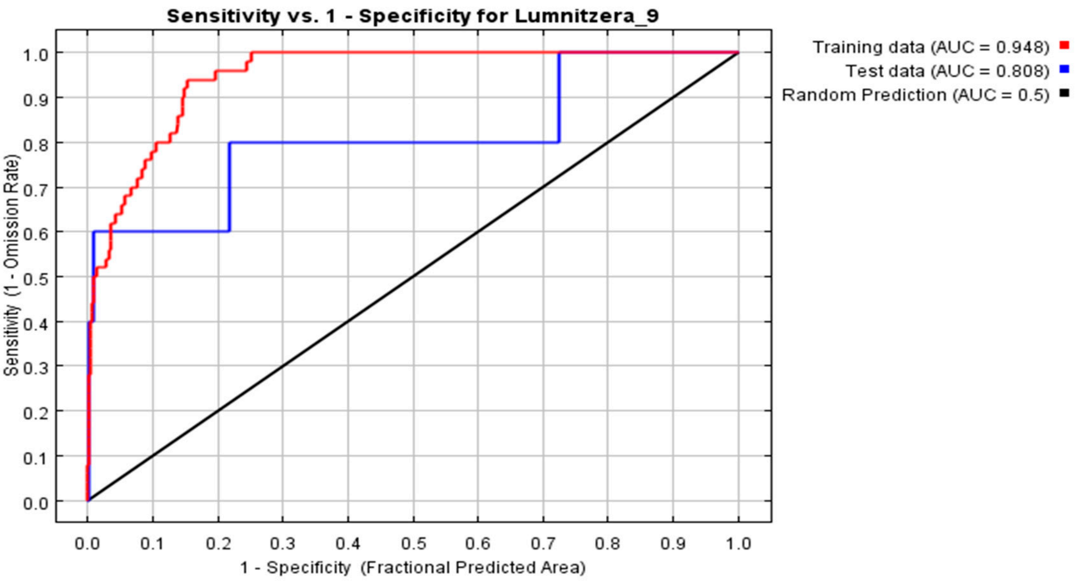

Figure S12. Validation results of ROC curve for the *Lumnitzera racemosa* prediction model (Optimum)

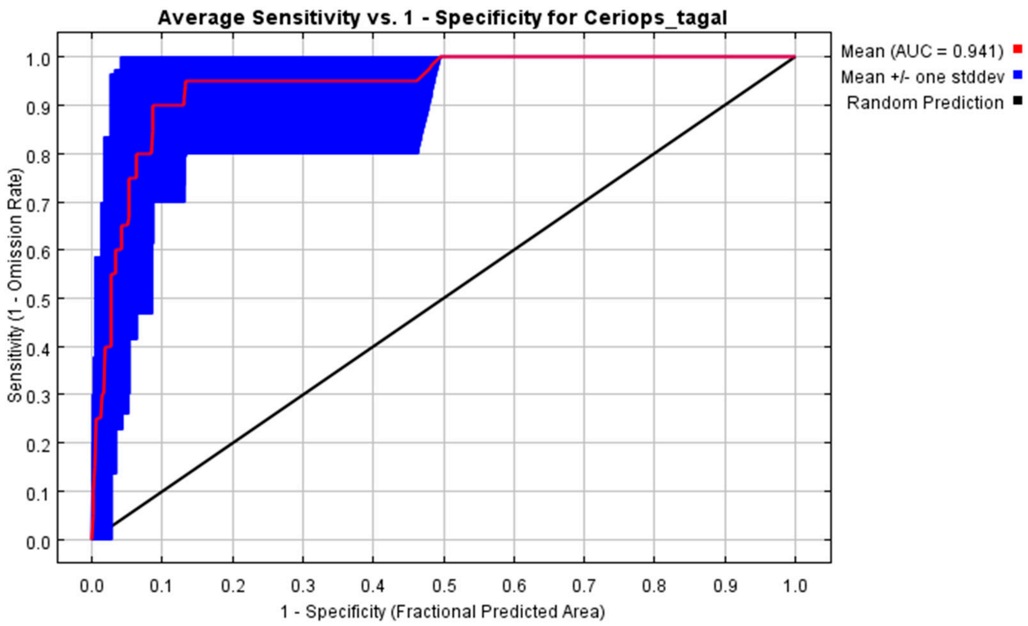

Figure S13 Validation results of ROC curve for the *Ceriops tagal* prediction model (Average)

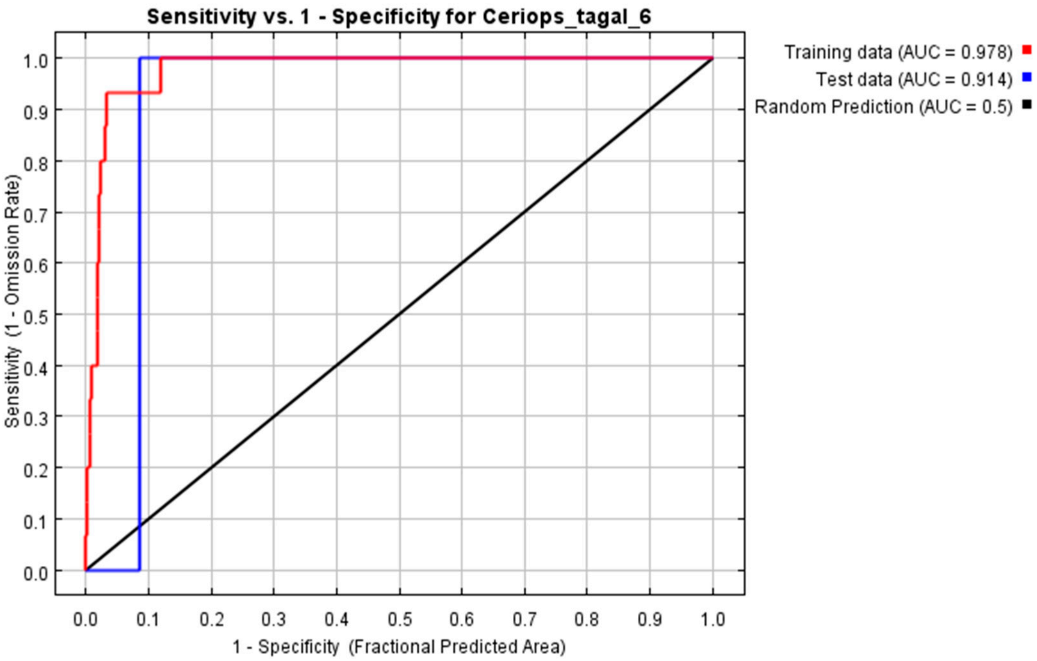

Figure S14. Validation results of ROC curve for the *Ceriops tagal* prediction model (Optimum)

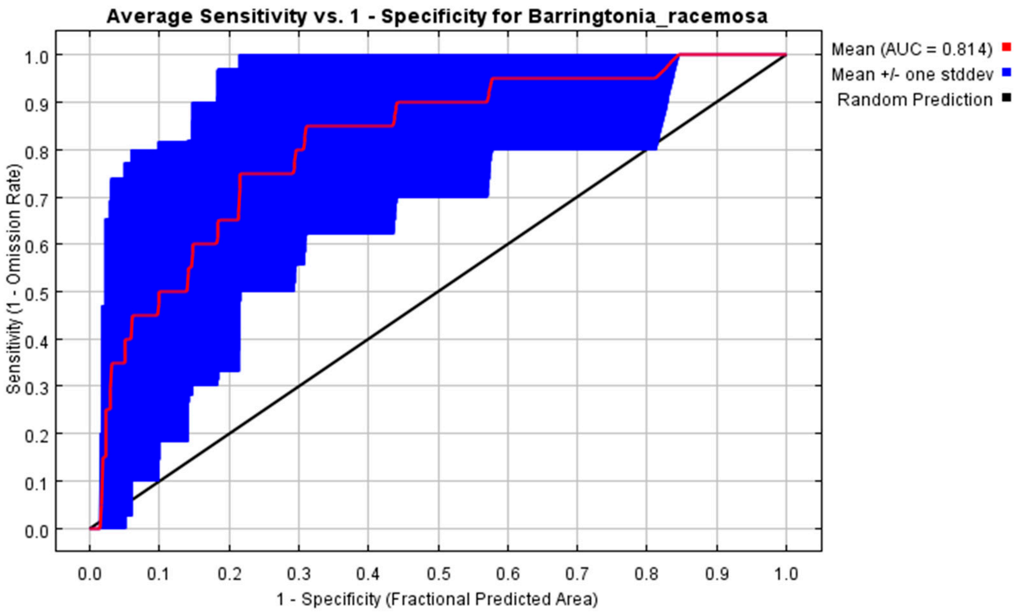

Figure S15. Validation results of ROC curve for the *Barringtonia racemosa* prediction model (Average)

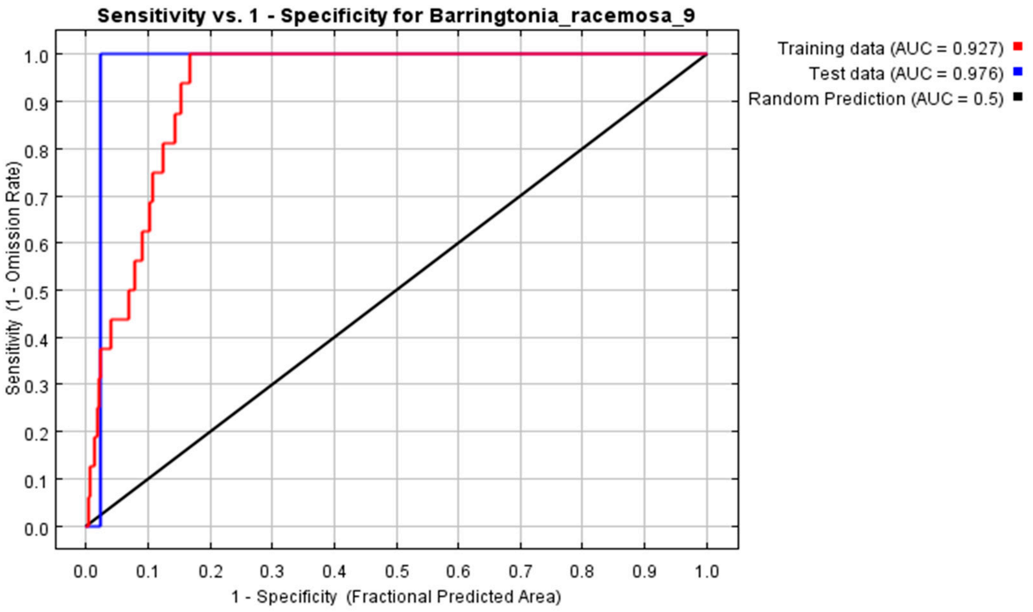

Figure S16. Validation results of ROC curve for the *Barringtonia racemosa* prediction model (Optimum)

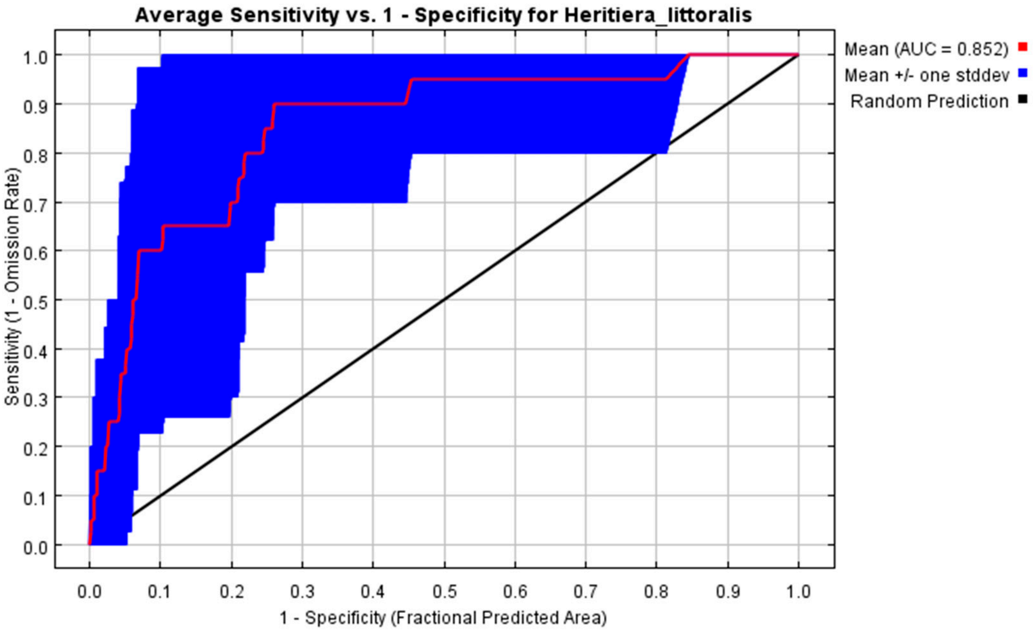

Figure S17. Validation results of ROC curve for the *Heritiera littoralis* prediction model (Average)

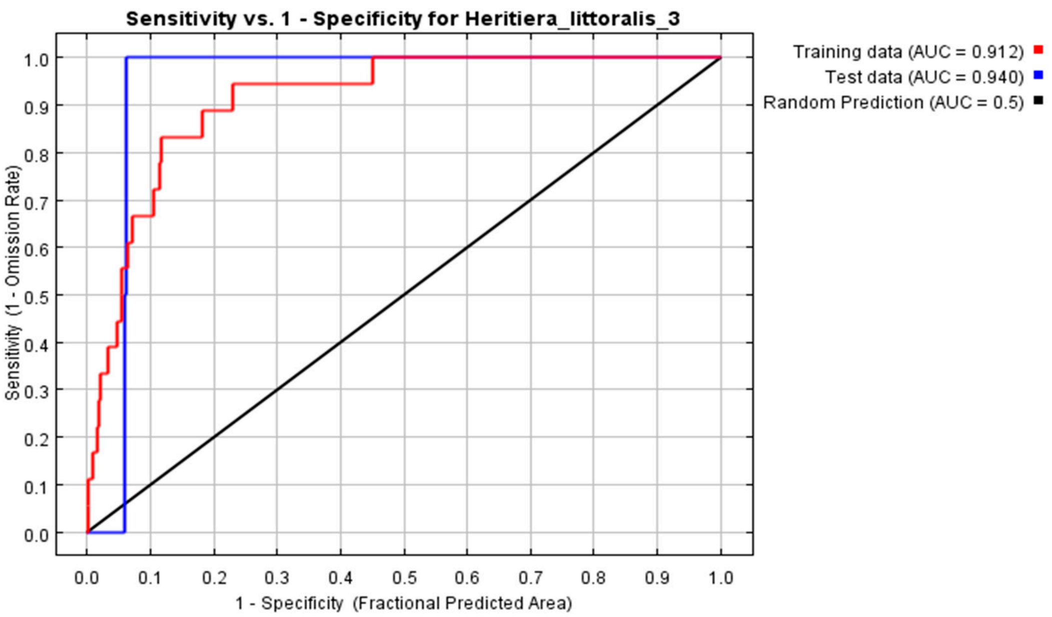

Figure S18. Validation results of ROC curve for the Heritiera littoralis prediction model (Optimum)

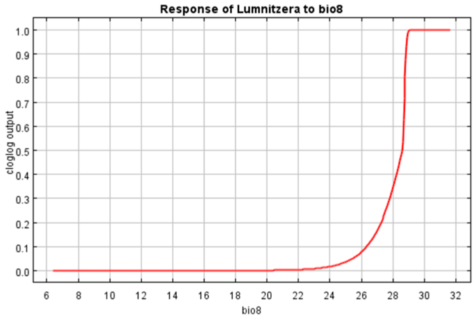

a. Wettest Quarter Mean Temperature

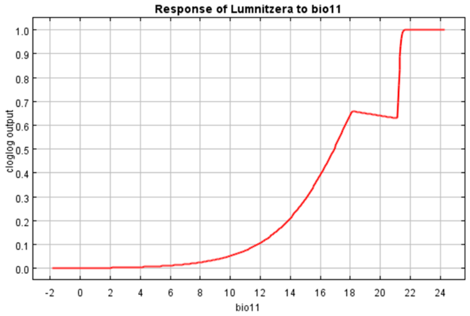

b. Coldest Quarter Mean Temperature

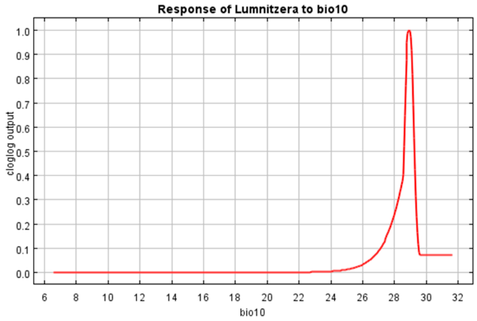

c. Warmest Quarter Mean Temperature

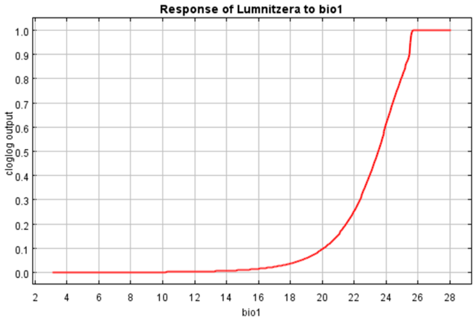

d. Annual Mean Temperature

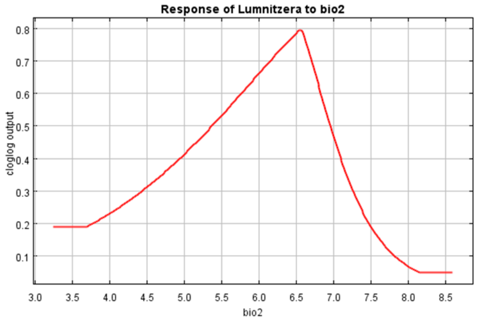

e. Mean Diurnal Range

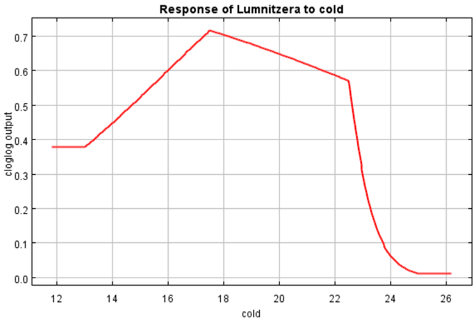

f. Average Sea Surface Temperature of Coldest Month

Figure S19. The main environmental factor response curves contributed by the *Lumnitzera racemosa* prediction model

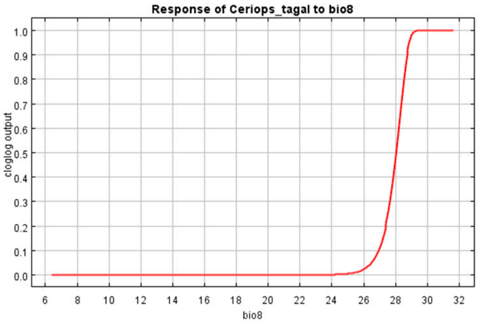

a. Wettest Quarter Mean Temperature

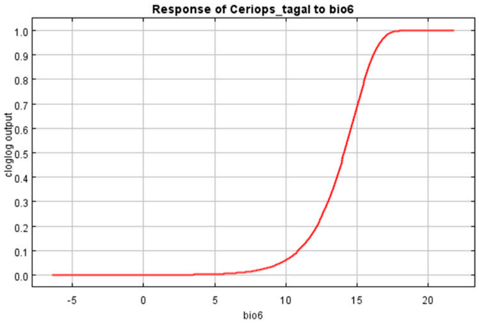

b. Minimum Temperature of Coldest Month

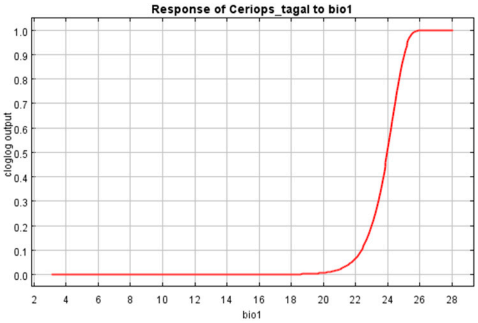

c. Annual Mean Temperature

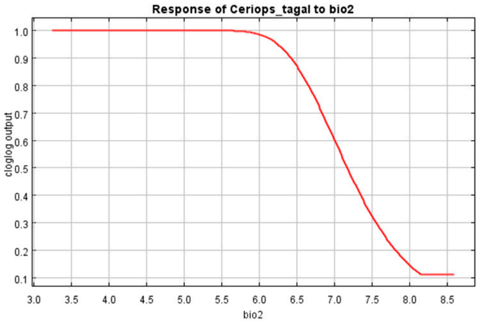

d. Mean Diurnal Range

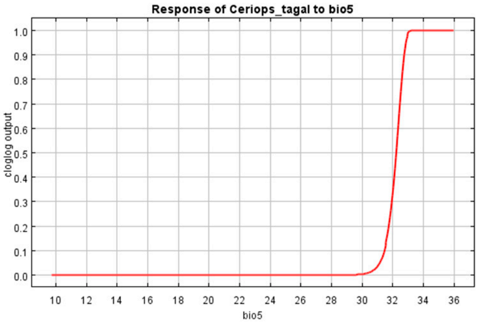

e. Maximum Temperature of Warmest Month

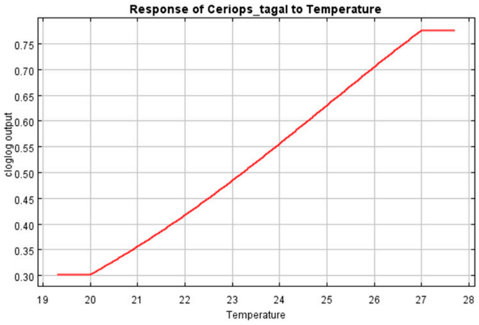

f. Mean Sea Surface Temperature

Figure S20. The main environmental factor response curves contributed by the *Ceriops tagal* prediction model

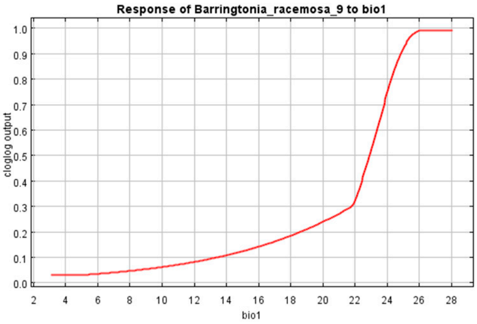

a. Annual Mean Temperature

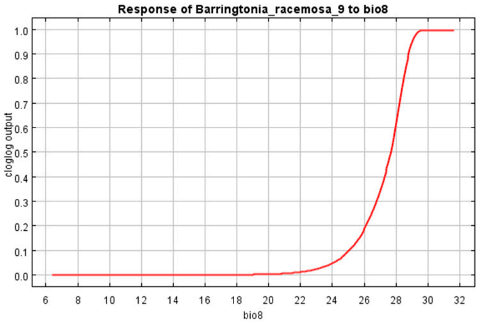

b. Wettest Quarter Mean Temperature

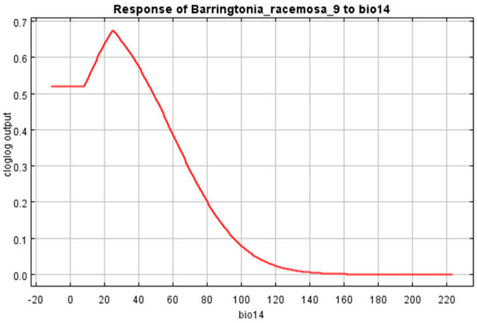

c. Precipitation of Driest Month

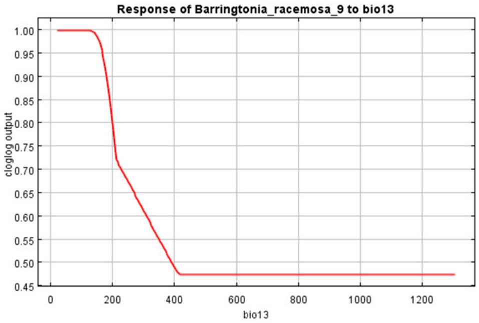

d. Precipitation of Wettest Month

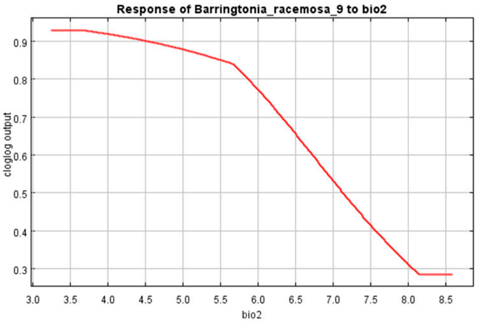

e. Mean Diurnal Range

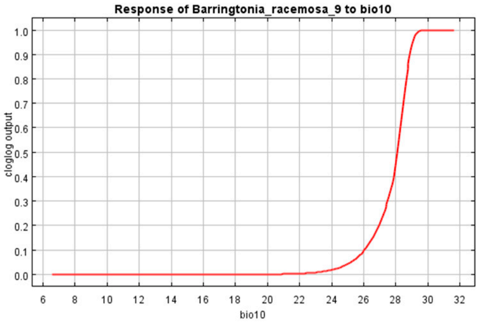

f. Warmest Quarter Mean Temperature

Figure S21. The main environmental factor response curves contributed by the *Barringtonia racemosa* prediction model

Table S1. Basic overview of mangrove sample plots in Guangdong, China

Species	Loc. Code	Sample Plot	Area (m^2)	Altitude (m)	Longitude (E)	Latitude (N)	Canopy (%)
Wild *Lumnitzera racemosa*	XW-L1	1-1	100	1	109.9350	20.2470	90
	XW-L1	1-2	100	2	109.9351	20.2471	90
	XW-L1	1-3	100	-1	109.9350	20.2472	93
	XW-L1	1-4	100	-3	109.9351	20.2471	86
	XW-L2	2-1	100	0	109.9257	20.2386	87
	XW-L2	2-2	100	3	109.9259	20.2368	91
	XW-L2	2-3	100	0	109.9260	20.2352	88
	XW-L2	2-4	100	3	109.9261	20.2336	92
	XW-L3	3-1	100	1	110.0000	20.3505	91
	XW-L3	3-2	100	0	110.0004	20.3507	85
Ceriops tagal	XW-G	JGM1-1	25	-1	110.0184	20.4147	85
	XW-G	JGM1-2	25	-2	110.0182	20.4147	83
	XW-G	JGM1-3	25	0	110.0184	20.4145	86
	XW-G	JGM1-4	25	-2	110.0182	20.4148	84
	XW-G	JGM1-5	25	-1	110.0181	20.4147	89
Barringtonia racemosa	LZ-JL	YR1-1	100	2	110.2858	20.6569	79
	LZ-JL	YR1-2	100	1	110.2862	20.6578	84
	LZ-JL	YR1-3	100	2	110.2860	20.6573	82
	SX-CR	YR2-1	100	-6	110.1383	21.1448	83
	SX-CR	YR2-2	100	-6	110.1384	21.1448	85
	SX-CR	YR2-3	100	2	110.1401	21.1479	83
	SX-CR	YR2-4	100	3	110.1399	21.1478	90
	SX-CR	YR2-5	100	1	110.1432	21.1697	84
Heritiera littoralis	LZ-JL	YYS1-1	NA	2	110.2861	20.6578	NA
	LJ-LD	YYS2-1	NA	-1	110.3836	21.4436	NA
	LJ-JL	YYS3-1	NA	5	110.3745	21.4297	NA
	MZ-CR	YYS4-1	NA	-4	110.1825	21.1111	NA

Note: XW-L1: Xipo Village, Jiaowei Township, Xuwen County (near County Road 698); XW-L2: Nanji Village, Jiaowei Township, Xuwen County; XW-L3: Maichen Town, Xuwen County (near Township Road 311); XW-G: Guancao Village, Maichen Town, Xuwen County (County Road 311)LZ-JL: Leizhou Jiulongshan Mangrove National Wetland ParkSX-CR: Chengyue River, Suixi CountyLJ-LD: Liangdong Village, Pote Town, Lianjiang CityLJ-JL: Jilongshan Mountain, Lianjiang CityMZ-CR: Chengyue River, Wenlili Village, Mazhang District

Table S2. Population characteristics of four mangrove species across different sampling sites.

Species	Site	Height (m)	Basal Diameter (cm)	Canopy Density (%)
Lumnitzera racemosa	Location 1	2.8±0.9	6.23±2.68	89.8%
Lumnitzera racemosa	Location 2	3.1±0.6	7.1±1.5	89.5%
Lumnitzera racemosa	Location 3	3.9±1.0	7.9±3.8	88.0%
Ceriops tagal	Maichen Town, Xuwen County	2.2±0.4	7.7±3.1	85.4%
Barringtonia racemosa	Site 1	4.0±0.9	14.5±6.1	81.6%
Barringtonia racemosa	Site 2	4.1±1.4	16.2±8.2	85.0%
Heritage Littoralis	Site 1	6.17±0.9	19.4±4.2	71.0%
Heritage Littoralis	Site 2	4.75±1.2	32.5±8.1	—
Heritage Littoralis	Site 3	5.1±2.5	25.9±12.1	74.0%
Heritage Littoralis	Site 4	4.4±1.8	26.1±9.9	—

Table S3. List of associated plants in the habitats of four mangrove and coastal tree species

Species	No.	Family	Genus	Scientific Name
Lumnitzera racemosa	1	Apocynaceae	*Strophanthus*	*Strophanthus divaricatus*
	2	Cordiaceae	*Cordia*	*Cordia dichotoma*
	3	Rutaceae	*Harrisonia*	*Harrisonia perforata*
	4	Phyllanthaceae	*Flueggea*	*Flueggea virosa*
	5	Phyllanthaceae	*Sauropus*	*Sauropus bacciformis*
	6	Salicaceae	*Flacourtia*	*Flacourtia indica*
	7	Amaranthaceae	*Achyranthes*	*Achyranthes aspera*
	8	Chenopodiaceae	*Suaeda*	*Suaeda australis*
	9	Aizoaceae	*Sesuvium*	*Sesuvium portulacastrum*
	10	Cactaceae	*Opuntia*	*Opuntia dillenii*
	11	Passifloraceae	*Passiflora*	*Passiflora foetida*
	12	Asparagaceae	*Agave*	*Agave sisalana*
	13	Ebenaceae	*Diospyros*	*Diospyros diversilimba*
	14	Ebenaceae	*Diospyros*	*Diospyros texana*
	15	Capparaceae	*Capparis*	*Capparis sepiaria*
	16	Cyperaceae	*Fimbristylis*	*Fimbristylis schoenoides*
	17	Moraceae	*Ficus*	*Ficus microcarpa*
	18	Moraceae	*Ficus*	*Ficus altissima*
	19	Oleaceae	*Jasminum*	*Jasminum nervosum*
	20	Casuarinaceae	*Casuarina*	*Casuarina equisetifolia*
	21	Verbenaceae	*Lantana*	*Lantana camara*
	22	Meliaceae	*Melia*	*Melia azedarach*
	23	Chenopodiaceae	*Salicornia*	*Salicornia europaea*
	24	Acanthaceae	*Avicennia*	*Avicennia marina*
	25	Asteraceae	*Symphyotrichum*	*Symphyotrichum subulatum*
	26	Asteraceae	*Pluchea*	*Pluchea indica*
	27	Asteraceae	*Bidens*	*Bidens pilosa*
	28	Asteraceae	*Chromolaena*	*Chromolaena odorata*
	29	Malvaceae	*Abutilon*	*Abutilon indicum*
	30	Malvaceae	*Abutilon*	*Abutilon theophrasti*
	31	Malvaceae	*Talipariti*	*Talipariti tiliaceum*
	32	Malvaceae	*Gossypium*	*Gossypium barbadense*
	33	Apocynaceae	*Catharanthus*	*Catharanthus roseus*
	34	Cucurbitaceae	*Melothria*	*Melothria pendula*
	35	Rhizophoraceae	*Bruguiera*	*Bruguiera gymnorhiza*
	36	Rhizophoraceae	*Rhizophora*	*Rhizophora stylosa*
	37	Poaceae	*Eleusine*	*Eleusine indica*
	38	Poaceae	*Dactyloctenium*	*Dactyloctenium aegyptium*
	39	Poaceae	*Cynodon*	*Cynodon dactylon*
	40	Pittosporaceae	*Pittosporum*	*Pittosporum tobira*
	41	Lygodiaceae	*Lygodium*	*Lygodium japonicum*
	42	Eriocaulaceae	*Eriocaulon*	*Eriocaulon buergerianum*
	43	Pteridaceae	*Acrostichum*	*Acrostichum aureum*
	44	Fabaceae	*Pongamia*	*Pongamia pinnata*
	45	Fabaceae	*Abrus*	*Abrus pulchellus*
	46	Fabaceae	*Caesalpinia*	*Caesalpinia bonduc*
	47	Lamiaceae	*Premna*	*Premna serratifolia*
	48	Lamiaceae	*Volkameria*	*Volkameria inermis*
Ceriops tagal	49	Acanthaceae	*Avicennia*	*Avicennia marina*
	50	Rhizophoraceae	*Rhizophora*	*Rhizophora stylosa*
	51	Primulaceae	*Aegiceras*	*Aegiceras corniculatum*
	52	Euphorbiaceae	*Excoecaria*	*Excoecaria agallocha*
	53	Asteraceae	*Chromolaena*	*Chromolaena odorata*
	54	Asteraceae	*Pluchea*	*Pluchea indica*
	55	Verbenaceae	*Lantana*	*Lantana camara*
	56	Asteraceae	*Bidens*	*Bidens pilosa*
	57	Lauraceae	*Litsea*	*Litsea glutinosa*
	58	Fabaceae	*Sesbania*	*Sesbania cannabina*
	59	Asteraceae	*Parthenium*	*Parthenium hysterophorus*
	60	Amaranthaceae	*Achyranthes*	*Achyranthes aspera*
	61	Chenopodiaceae	*Salicornia*	*Salicornia europaea*
	62	Poaceae	*Dactyloctenium*	*Dactyloctenium aegyptium*
	63	Meliaceae	*Melia*	*Melia azedarach*
	64	Lamiaceae	*Volkameria*	*Volkameria inermis*
	65	Combretaceae	*Lumnitzera*	*Lumnitzera racemosa*
	66	Asteraceae	*Praxelis*	*Praxelis clematidea*

Species	No.	Family	Genus	Scientific Name
	67	Verbenaceae	*Stachytarpheta*	*Stachytarpheta jamaicensis*
Barringtonia racemosa	68	Malvaceae	*Talipariti*	*Talipariti tiliaceum*
	69	Arecaceae	*Caryota*	*Caryota mitis*
	70	Lauraceae	*Litsea*	*Litsea glutinosa*
	71	Rutaceae	*Psilopeganum*	*Psilopeganum sinense*
	72	Rutaceae	*Melicope*	*Melicope pteleifolia*
	73	Phyllanthaceae	*Aporosa*	*Aporosa dioica*
	74	Melastomataceae	*Melastoma*	*Melastoma candidum*
	75	Convolvulaceae	*Ipomoea*	*Ipomoea triloba*
	76	Flagellariaceae	*Flagellaria*	*Flagellaria indica*
	77	Amaranthaceae	*Amaranthus*	*Amaranthus viridis*
	78	Amaranthaceae	*Achyranthes*	*Achyranthes aspera*
	79	Amaranthaceae	*Alternanthera*	*Alternanthera philoxeroides*
	80	Araliaceae	*Heptapleurum*	*Heptapleurum heptaphyllum*
	81	Araliaceae	*Aralia*	*Aralia finlaysoniana*
	82	Araceae	*Alocasia*	*Alocasia odora*
	83	Clusiaceae	*Garcinia*	*Garcinia hanburyi*
	84	Amaryllidaceae	*Crinum*	*Crinum asiaticum*
	85	Moraceae	*Ficus*	*Ficus auriculata*
	86	Rosaceae	*Rubus*	*Rubus leucanthus*
	87	Rubiaceae	*Paederia*	*Paederia foetida*
	88	Lythraceae	*Sonneratia*	*Sonneratia apetala*
	89	Verbenaceae	*Lantana*	*Lantana camara*
	90	Basellaceae	*Basella*	*Basella alba*
	91	Pandanaceae	*Pandanus*	*Pandanus tectorius*
	92	Meliaceae	*Melia*	*Melia azedarach*
	93	Acanthaceae	*Acanthus*	*Acanthus ilicifolius*
	94	Acanthaceae	*Ruellia*	*Ruellia simplex*
	95	Asteraceae	*Parthenium*	*Parthenium hysterophorus*
	96	Asteraceae	*Wollastonia*	*Wollastonia biflora*
	97	Asteraceae	*Bidens*	*Bidens pilosa*
	98	Asteraceae	*Chromolaena*	*Chromolaena odorata*
	99	Malvaceae	*Heritiera*	*Heritiera littoralis*
	100	Malvaceae	*Corchorus*	*Corchorus aestuans*
	101	Malvaceae	*Sterculia*	*Sterculia lanceolata*
	102	Malvaceae	*Talipariti*	*Talipariti tiliaceum*
	103	Thelypteridaceae	*Cyclosorus*	*Cyclosorus interruptus*
	104	Piperaceae	*Piper*	*Piper sarmentosum*
	105	Poaceae	*Bambusa*	*Bambusa vulgaris*
	106	Poaceae	*Phragmites*	*Phragmites australis*
	107	Poaceae	*Isachne*	*Isachne globosa*
	108	Poaceae	*Digitaria*	*Digitaria setigera*
	109	Pteridaceae	*Acrostichum*	*Acrostichum aureum*
	110	Menispermaceae	*Tinospora*	*Tinospora sinensis*
	111	Fabaceae	*Derris*	*Derris trifoliata*
	112	Fabaceae	*Pongamia*	*Pongamia pinnata*
	113	Fabaceae	*Pueraria*	*Pueraria montana*
	114	Euphorbiaceae	*Excoecaria*	*Excoecaria agallocha*
	115	Euphorbiaceae	*Mallotus*	*Mallotus paniculatus*
	116	Lamiaceae	*Volkameria*	*Volkameria inermis*
	117	Lamiaceae	*Clerodendrum*	*Clerodendrum cyrtophyllum*
	118	Musaceae	*Musa*	*Musa basjoo*
	119	Rhizophoraceae	*Bruguiera*	*Bruguiera gymnorhiza*
Heritiera littoralis	120	Lecythidaceae	*Barringtonia*	*Barringtonia racemosa*
	121	Lauraceae	*Litsea*	*Litsea glutinosa*
	122	Rutaceae	*Acronychia*	*Acronychia pedunculata*
	123	Rutaceae	*Melicope*	*Melicope pteleifolia*
	124	Rutaceae	*Clausena*	*Clausena lansium*
	125	Rutaceae	*Atalantia*	*Atalantia buxifolia*
	126	Cannabaceae	*Celtis*	*Celtis timorensis*
	127	Phyllanthaceae	*Aporosa*	*Aporosa dioica*
	128	Phyllanthaceae	*Phyllanthus*	*Phyllanthus urinaria*
	129	Phyllanthaceae	*Bridelia*	*Bridelia tomentosa*
	130	Commelinaceae	*Commelina*	*Commelina diffusa*
	131	Convolvulaceae	*Ipomoea*	*Ipomoea triloba*
	132	Convolvulaceae	*Ipomoea*	*Ipomoea pes-caprae*
	133	Flagellariaceae	*Flagellaria*	*Flagellaria indica*

Species	No.	Family	Genus	Scientific Name
	134	Amaranthaceae	*Achyranthes*	*Achyranthes aspera*
	135	Amaranthaceae	*Alternanthera*	*Alternanthera philoxeroides*
	136	Passifloraceae	*Passiflora*	*Passiflora foetida*
	137	Pentaphylacaceae	*Eurya*	*Eurya loquaiana*
	138	Araliaceae	*Heptapleurum*	*Heptapleurum heptaphyllum*
	139	Sapindaceae	*Arytera*	*Arytera littoralis*
	140	Myrtaceae	*Syzygium*	
	141	Myrtaceae	*Psidium*	*Psidium guajava*
	142	Dioscoreaceae	*Dioscorea*	*Dioscorea opposita*
	143	Rhamnaceae	*Sageretia*	*Sageretia thea*
	144	Symplocaceae	*Symplocos*	*Symplocos sumuntia*
	145	Moraceae	*Ficus*	*Ficus microcarpa*
	146	Moraceae	*Ficus*	*Ficus benjamina*
	147	Moraceae	*Ficus*	*Ficus subpisocarpa*
	148	Rosaceae	*Photinia*	*Photinia prunifolia*
	149	Rubiaceae	*Mussaenda*	*Mussaenda pubescens*
	150	Rubiaceae	*Benkara*	*Benkara scandens*
	151	Rubiaceae	*Ixora*	*Ixora chinensis*
	152	Rubiaceae	*Spermacoce*	*Spermacoce alata*
	153	Rubiaceae	*Psychotria*	*Psychotria asiatica*
	154	Rubiaceae	*Paederia*	*Paederia foetida*
	155	Lythraceae	*Sonneratia*	*Sonneratia apetala*
	156	Anacardiaceae	*Lannea*	*Lannea coromandelica*
	157	Oleaceae	*Olea*	*Olea brachiata*
	158	Oleaceae	*Jasminum*	*Jasminum nervosum*
	159	Melastomataceae	*Melastoma*	*Melastoma candidum*
	160	Acanthaceae	*Avicennia*	*Avicennia marina*
	161	Basellaceae	*Basella*	*Basella alba*
	162	Pandanaceae	*Pandanus*	*Pandanus tectorius*
	163	Pteridaceae	*Acrostichum*	*Acrostichum aureum*
	164	Meliaceae	*Melia*	*Melia azedarach*
	165	Acanthaceae	*Acanthus*	*Acanthus ilicifolius*
	166	Acanthaceae	*Ruellia*	*Ruellia simplex*
	167	Asteraceae	*Cyanthillium*	*Cyanthillium cinereum*
	168	Asteraceae	*Mikania*	*Mikania micrantha*
	169	Aasteraceae	*Pluchea*	*Pluchea indica*
	170	Asteraceae	*Synedrella*	*Synedrella nodiflora*
	171	Asteraceae	*Bidens*	*Bidens pilosa*
	172	Malvaceae	*Microcos*	*Microcos paniculata*
	173	Malvaceae	*Sterculia*	*Sterculia lanceolata*
	174	Malvaceae	*Talipariti*	*Talipariti tiliaceum*
	175	Malvaceae	*Urena*	*Urena lobata*
	176	Apocynaceae	*Cerbera*	*Cerbera manghas*
	177	Rhizophoraceae	*Bruguiera*	*Bruguiera gymnorhiza*
	178	Poaceae	*Phragmites*	*Phragmites australis*
	179	Poaceae	*Isachne*	*Isachne globosa*
	180	Lygodiaceae	*Lygodium*	*Lygodium japonicum*
	181	Pteridaceae	*Acrostichum*	*Acrostichum speciosum*
	182	Caricaceae	*Carica*	*Carica papaya*
	183	Annonaceae	*Uvaria*	*Uvaria macrophylla*
	184	Elaeocarpaceae	*Elaeocarpus*	*Elaeocarpus sylvestris*
	185	Fabaceae	*Canavalia*	*Canavalia ensiformis*
	186	Fabaceae	*Pongamia*	*Pongamia pinnata*
	187	Fabaceae	*Archidendron*	*Archidendron lucidum*

Table S4. Static life tables for mangrove species populations in Leizhou Peninsula

Species	Age Class	Diameter Class (cm)	a_x	l_x	lnl_x	d_x	q_x	L_x	T_x	e_x	S_x	F_x	K_x
Lumnitzera racemosa	I	D_{BH} ≤ 1	28	1000	6.91	-2893	-2.89	2446.43	11428.57	11.43	3.89	-2.89	-1.36
	II	1 < D_{BH} ≤ 4	109	3893	8.27	464	0.12	3660.71	8982.14	2.31	0.88	-2.43	0.13
	III	4 < D_{BH} ≤ 7	96	3429	8.14	1036	0.3	2910.71	5321.43	1.55	0.7	-1.39	0.36
	IV	7 < D_{BH} ≤ 10	67	2393	7.78	1607	0.67	1589.29	2410.71	1.01	0.33	0.21	1.11
	V	10 < D_{BH} ≤ 13	22	786	6.67	536	0.68	517.86	821.43	1.05	0.32	0.75	1.15
	VI	13 < D_{BH} ≤ 16	7	250	5.52	107	0.43	196.43	303.57	1.21	0.57	0.86	0.56
	VII	16 < D_{BH} ≤ 19	4	143	4.96	107	0.75	89.29	107.14	0.75	0.25	0.96	1.39
	VIII	D_{BH} > 19	1	36	3.58	—	—	17.86	17.86	0.50	—	—	—
Ceriops tagal	I	D_{BH}≤1	1411	1000	6.91	980	0.98	509.92	592.84	0.59	0.02	0.98	3.92
	II	1 < D_{BH}≤4	28	20	2.99	-28	-1.39	33.66	82.92	4.18	2.39	0.95	-0.87
	III	4 < D_{BH}≤7	67	47	3.86	30	0.64	32.25	49.26	1.04	0.36	0.98	1.03
	IV	7 < D_{BH}≤10	24	17	2.83	11	0.67	11.34	17.01	1.00	0.33	0.99	1.10
	V	10 < D_{BH}≤13	8	6	1.74	4	0.75	3.54	5.67	1.00	0.25	1.00	1.39
	VI	13 < D_{BH}≤16	2	1	0.35	1	0.50	1.06	2.13	1.50	0.50	1.00	0.69
	VII	16 < D_{BH}≤19	1	1	-0.34	0	0.00	0.71	1.06	1.50	1.00	1.00	0.00
	VIII	19 < D_{BH}	1	1	-0.34	—	—	0.35	0.35	0.50	—	1.00	—
Barringtonia racemosa	Ⅰ	D_{BH}≤1	83	1000	6.91	494	0.49	753.01	3620.48	3.62	0.51	0.49	0.68
	Ⅱ	1 < D_{BH}≤4	42	506	6.23	193	0.38	409.64	2867.47	5.67	0.62	0.69	0.48
	Ⅲ	4 < D_{BH}≤7	26	313	5.75	-84	-0.27	355.42	2457.83	7.85	1.27	0.60	-0.24
	Ⅳ	7 < D_{BH}≤10	33	398	5.99	-72	-0.18	433.73	2102.41	5.29	1.18	0.53	-0.17
	Ⅴ	10 < D_{BH}≤13	39	470	6.15	48	0.10	445.78	1668.67	3.55	0.90	0.58	0.11
	Ⅵ	13 < D_{BH}≤16	35	422	6.04	0	0.00	421.69	1222.89	2.90	1.00	0.58	0.00
	Ⅶ	16 < D_{BH}≤19	35	422	6.04	169	0.40	337.35	801.20	1.90	0.60	0.75	0.51
	Ⅷ	19 < D_{BH}≤22	21	253	5.53	60	0.24	222.89	463.86	1.83	0.76	0.81	0.27
	Ⅸ	22 < D_{BH}≤25	16	193	5.26	145	0.75	120.48	240.96	1.25	0.25	0.95	1.39
	Ⅹ	25 < D_{BH}≤28	4	48	3.88	-48	-1.00	72.29	120.48	2.50	2.00	0.90	-0.69
	Ⅺ	28 < D_{BH}	8	96	4.57	—	—	48.19	48.19	0.50	—	1.00	—
Heritiera littoralis	Ⅰ	D_{BH}≤5	3	1000	6.91	0	0.00	1000.00	14166.67	14.17	1.00	0.00	0.00
	Ⅱ	5 < D_{BH}≤10	3	1000	6.91	-333	-0.33	1166.67	13166.67	13.17	1.33	-0.33	-0.29
	Ⅲ	10 < D_{BH}≤15	4	1333	7.20	-1333	-1.00	2000.00	12000.00	9.00	2.00	-1.67	-0.69
	Ⅳ	15 < D_{BH}≤20	8	2667	7.89	1000	0.38	2166.67	10000.00	3.75	0.63	-0.67	0.47
	Ⅴ	20 < D_{BH}≤25	5	1667	7.42	-333	-0.20	1833.33	7833.33	4.70	1.20	-1.00	-0.18
	Ⅵ	25 < D_{BH}≤30	6	2000	7.60	667	0.33	1666.67	6000.00	3.00	0.67	-0.33	0.41
	Ⅶ	30 < D_{BH}≤35	4	1333	7.20	-2000	-1.50	2333.33	4333.33	3.25	2.50	-2.33	-0.92
	Ⅷ	35 < D_{BH}≤40	10	3333	8.11	3000	0.90	1833.33	2000.00	0.60	0.10	0.67	2.30
	Ⅸ	D_{BH} > 40	1	333	5.81	NA	NA	166.67	166.67	0.50	—	1.00	—

Note：D_{BH}: Diameter at Breast Height; a_x：Current survival quantity at age x level； l_x：Standard survival quantity at the beginning of age level x ； lnl_x：Normalized survival logarithm of l_x； d_x：Standard number of deaths between x and x+1 age groups ； q_x：Period mortality rate between x and x+1 age groups； L_x：The average number of surviving individuals between age groups x to x+1； T_x：Total number of standard individuals at age level x and above； e_x：The average expectation of individual plants at age x； S_x：Population survival rate； F_x：Cumulative mortality rate； K_x：Disappearance rate；“—” Unable to calculate and obtain numerical value.

Table S5. Population dynamic indices (V_n, V_{pi}, V'_{pi}, P_{max}) across age-class transitions for four mangrove species

Species	Age Class	Dynamic Index (V_n) (%)	V_{pi} (%)	V'_{pi} (%)	P_{max}
Lumnitzera racemosa	I-II	-74.31	26.18	3.273	0.125
	II-III	11.93			
	III-IV	30.21			
	IV-V	67.16			
	V-VI	68.18			
	VI-VII	42.86			
	VII-VIII	75			
Ceriops tagal	I-II	98.04	92.99	11.624	0.125
	II-III	-58.21			
	III-IV	64.18			
	IV-V	66.67			
	V-VI	75			
	VI-VII	50			
	VII-VIII	0			
Barringtonia racemosa	I ~ II	49.40	23.77	0.547	0.023
	II ~ III	38.10			
	III ~ IV	-21.21			
	IV ~ V	-15.38			
	V ~ VI	10.26			
	VI ~ VII	0.00			
Heritiera littoralis	I ~ II	0.00	18.63	2.068	0.111
	II ~ III	-25.00			
	III ~ IV	-50.00			
	IV ~ V	37.50			
	V ~ VI	-16.70			
	VI ~ VII	33.30			
	VII ~ VIII	-60.00			
	VIII ~ IX	90.00			

Table S6. Dynamic time-series table of population quantities for four mangrove species

Species	Age Class	Initial Number	M_t			
			M_2	M_4	M_6	M_8
Lumnitzera racemosa	I	28	—	—	—	—
	II	109	68.5	—	—	—
	III	96	102.5	—	—	—
	IV	67	81.5	75	—	—
	V	22	44.5	73.5	—	—
	VI	7	14.5	48	55	—
	VII	4	5.5	25	51	—
	VIII	1	2.5	8.5	33	42
Ceriops tagal	I	1411	—	—	—	—
	II	28	719.5	—	—	—
	III	67	47.5	—	—	—
	IV	24	45.5	382.5	—	—
	V	8	16	32	—	—
	VI	2	5	25	257	—
	VII	1	1.5	9	22	—
	VIII	1	1	3	17	193
Barringtonia racemosa	I	83	—	—	—	—
	II	42	62.5	—	—	—
	III	26	34	—	—	—
	IV	33	29.5	46	—	—
	V	39	36	35	—	—
	VI	35	37	33.25	43	—
	VII	35	35	35.5	35	—
	VIII	21	28	32.5	32	39
	IX	16	18.5	26.75	30	31
	X	4	10	19	25	26
	XI	8	6	12.25	20	24
Heritiera littoralis	I	3	—	—	—	—
	II	3	3	—	—	—
	III	4	3.5	—	—	—
	IV	8	6	4.5	—	—
	V	5	6.5	5	—	—
	VI	6	5.5	5.75	5	—
	VII	4	5	5.75	5	—
	VIII	10	7	6.25	6	5
	IX	1	5.5	5.25	6	5

Note: M_t represents the number of individuals in each age group after n future age groups. "—" has no corresponding value.

Table S7. Consolidated geographical distribution data of four mangrove species

Species	Longitude (°E)	Latitude (°N)
Lumnitzera racemosa	109.93	20.23
	109.93	20.23
	109.93	20.24
	109.93	20.23
	110.00	20.35
	109.93	20.26
	109.93	20.26
	109.98	20.28
	110.01	20.27
	110.03	20.29
	109.95	20.44
	110.02	20.42
	110.03	20.45
	109.99	20.38
	109.99	20.37
	109.99	20.34
	109.98	20.35
	109.98	20.35
	109.99	20.35
	109.99	20.34
	109.99	20.35
	118.15	24.52
	118.13	24.53
	118.07	24.45
	114.22	22.46
	117.88	24.12
	110.03	20.42
	109.51	18.24
	108.36	21.69
	110.39	20.01
	110.59	19.96
	108.25	21.63
	109.94	20.25
	109.94	20.25
	109.94	20.25
	109.51	18.25
	110.20	20.40
	120.70	23.00
	117.70	24.00
	108.08	21.53
	109.55	19.85
	109.72	18.25
	110.60	20.00
	113.62	22.42
	109.75	21.50
	110.80	19.90
	109.62	18.23
	110.20	20.30
	110.80	19.60
	110.40	20.00
	110.40	18.80
	108.40	21.80
	109.20	21.70
	108.60	19.10
	109.70	19.20
	120.10	23.03
	108.19	21.65
	110.59	20.02

Continued Table

Species	Longitude (°E)	Latitude (°N)
	108.12	21.53
	110.83	19.63
	109.70	18.26
	109.72	18.33
	109.62	18.23
	109.62	18.22
	109.61	18.22
	109.51	18.25
	109.11	18.37
	109.57	18.26
	114.90	22.62
	114.95	22.73
	114.97	22.70
	115.05	22.77
	108.30	21.55
Ceriops tagal	110.50	19.77
	110.21	20.44
	120.31	22.63
	110.35	20.00
	110.17	20.33
	110.80	19.55
	109.55	19.85
	109.62	18.23
	110.86	19.64
	110.06	19.97
	110.63	19.89
	110.02	20.41
	109.28	19.74
	110.54	20.00
	109.98	20.29
	110.06	20.29
Barringtonia racemosa	110.14	21.15
	110.14	21.15
	110.29	20.66
	110.14	21.46
	110.14	21.16
	110.14	21.17
	110.60	19.95
	113.62	22.42
	120.46	22.65
	110.40	18.80
	121.14	22.76
	118.09	24.45
	110.32	18.67
	114.01	22.54
	118.12	24.45
	110.76	19.63
	110.35	20.65
	109.38	19.76
	110.84	19.56
	110.91	19.67
Heritiera littoralis	118.09	24.45
	114.52	22.65
	114.60	22.61
	114.52	22.65
	114.28	22.64
	108.19	21.65
	110.79	19.63
	108.13	21.56
	110.29	20.66
	110.38	21.44
	110.37	21.43
	110.18	21.11
	113.65	22.42
	114.00	22.53
	120.83	22.17
	114.02	22.53
	108.26	21.60

Species	Longitude (°E)	Latitude (°N)
	108.12	21.54
	110.60	19.97
	110.82	19.92
	114.27	22.73
	113.36	23.19

Table S8. Contribution percentages of environmental variables in species distribution prediction models

Variable Code	Variable Description	*Lumnitzera racemosa*	*Ceriops tagal*	*Barringtonia racemosa*	*Heritiera littoralis*
Temperature Variables					
bio1	Annual Mean Temperature	11.3	20.2	37.8	-
bio2	Mean Diurnal Range	6.1	4.8	3.9	31.1
bio3	Isothermality	1.1	1.1	1.5	-
bio4	Temperature Seasonality (SD)	0.3	3.1	-	2
bio5	Max Temperature of Warmest Month	0.7	4	0.1	-
bio6	Min Temperature of Coldest Month	1.3	24.6	1.2	-
bio7	Temperature Annual Range	-	-	-	0.2
bio8	Mean Temperature of Wettest Quarter	32.3	-	35.4	46.5
bio9	Mean Temperature of Driest Quarter	-	-	0.7	0.1
bio10	Mean Temperature of Warmest Quarter	12.4	0.9	3.7	0.2
bio11	Mean Temperature of Coldest Quarter	17.2	1.4	-	-
Precipitation Variables					
bio12	Annual Precipitation	0.6	0.1	-	0.5
bio13	Precipitation of Wettest Month	4	0.2	4.2	0
bio14	Precipitation of Driest Month	-	1.2	7	11.4
bio15	Precipitation Seasonality (CV)	0.3	0.7	0	0.7
bio16	Precipitation of Wettest Quarter	-	-	3.5	0.4
bio17	Precipitation of Driest Quarter	2.5	-	-	0
bio18	Precipitation of Warmest Quarter	0	-	-	4.9
bio19	Precipitation of Coldest Quarter	3.8	0.2	0.9	1.5
Marine Variables					
Temperature	Mean Sea Surface Temperature	1.3	3.3	0.1	-
Cold	SST of Coldest Month	4.3	-	-	-
Salinity	Mean Sea Surface Salinity	0.5	1.4	-	-
range salinity	Annual Sea Surface Salinity Range	-	-	-	0.5

Note：*All variables derived from WorldClim BIO dataset except marine parameters. Contribution percentages represent relative importance in MaxEnt species distribution models. SST = Sea Surface Temperature.*

Table S9. Environmental variables dataset

Variable cod	Variable Description	Unit
bio1	Annual Mean Temperature	℃
bio2	Mean Diurnal Range	℃
bio3	Isothermality	%
bio4	Temperature Seasonality	℃
bio5	Max Temperature of Warmest Month	℃
bio6	Min Temperature of Coldest Month	℃
bio7	Temperature Annual Range	℃
bio8	Mean Temperature of Wettest Quarter	℃
bio9	Mean Temperature of Driest Quarter	℃
bio10	Mean Temperature of Warmest Quarter	℃
bio11	Mean Temperature of Coldest Quarter	℃
bio12	Annual Precipitation	mm
bio13	Precipitation of Wettest Month	mm
bio14	Precipitation of Driest Month	mm
bio15	Precipitation Seasonality (Coefficient of Variation)	mm
bio16	Precipitation of Wettest Quarter	mm
bio17	Precipitation of Driest Quarter	mm
bio18	Precipitation of Warmest Quarter	mm
bio19	Precipitation of Coldest Quarter	mm
hot	Average sea surface temperature in the hottest month	℃
cold	Average Sea Surface Temperature of Coldest Month	℃
Temperature	Mean sea surface temperature	℃
salinity	Average sea surface salinity	‰
range salinity	Annual average sea surface salinity variation range	‰
